# Reading cognition from the eyes: association of retinal nerve fibre layer thickness with cognitive performance in a population-based study

**DOI:** 10.1093/braincomms/fcab258

**Published:** 2021-11-08

**Authors:** Johanna Girbardt, Tobias Luck, Jana Kynast, Francisca S Rodriguez, Barbara Wicklein, Kerstin Wirkner, Christoph Engel, Christian Girbardt, Mengyu Wang, Maryna Polyakova, A Veronica Witte, Markus Loeffler, Arno Villringer, Steffi G Riedel-Heller, Matthias L Schroeter, Tobias Elze, Franziska G Rauscher

**Affiliations:** 1 Institute for Medical Informatics, Statistics and Epidemiology, Leipzig University, 04107 Leipzig, Germany; 2 Department of Neurology, Max Planck Institute for Human Cognitive and Brain Sciences, 04103 Leipzig, Germany; 3 Faculty of Applied Social Sciences, University of Applied Sciences Erfurt, 99085 Erfurt, Germany; 4 Institute of Social Medicine, Occupational Health and Public Health (ISAP), Leipzig University, 04103 Leipzig, Germany; 5 German Centre for Neurodegenerative Diseases (DZNE), Research Group Psychosocial Epidemiology and Public Health, 17489 Greifswald, Germany; 6 Leipzig Research Centre for Civilization Diseases (LIFE), Leipzig University, 04103 Leipzig, Germany; 7 Department of Ophthalmology, Leipzig University Medical Center, 04103 Leipzig, Germany; 8 Schepens Eye Research Institute, Harvard Medical School, Boston, MA 02114, USA; 9 Clinic of Cognitive Neurology, Leipzig University Medical Center, 04103 Leipzig, Germany

**Keywords:** retinal nerve fibre layer, cognition, dementia, the Consortium to Establish a Registry for Alzheimer’s disease (CERAD), population-based

## Abstract

With the eye as a window to the brain, non-invasive fast screening of retinal nerve fibre layer thickness poses the opportunity for early detection of cognitive decline leading to dementia. Our objective is to determine whether performance in various neurocognitive tests has an association with itemized retinal nerve fibre layer thickness. Detailed investigation of associations factored in sex and eye-side. The large population-based LIFE-Adult study (Leipzig Research Centre for Civilization Diseases) was conducted at Leipzig University, Germany from 2011 to 2014. Randomly selected participants (*N* = 10 000) were drawn from population registry in an age- and gender-stratified manner, focusing on 40–80 years. Cognitive function was examined with the CERAD-NP Plus test-battery (Consortium to Establish a Registry for Alzheimer’s Disease), Stroop-Test, Reading the Mind in the Eyes-Test and Multiple-Choice Vocabulary Intelligence Test. Circumpapillary retinal nerve fibre layer thickness was measured with Optical Coherence Tomography. Subjects with reliable measurements (≥50 B-scan repetitions, signal-to-noise-ratio ≥20 dB, ≤5% missing A-scans) and without clinical eye pathology (sample A) and additional exclusion due to conditions of the central nervous system (sample B) were evaluated. The relationship between cognitive function and retinal nerve fibre layer thickness was investigated for six segments: temporal, temporal-superior, temporal-inferior, nasal, nasal-superior and nasal-inferior. For comparison with other studies, global mean is given. Brain-side projection analysis links results to the corresponding brain hemisphere. We analysed 11 124 eyes of 6471 subjects [55.5 years of age (19.1–79.8 years), 46.9% male]. Low cognitive performance was predominantly associated with thinner retinal nerve fibre layer thickness. Correlation analysis indicated emphasis on global and temporally located effects. Multivariable regression analysis with adjustments (age, sex and scan radius) presented individual results for each test, differentiating between sex and eye-side. For instance, verbal fluency tests and Trail Making Test-B show stronger association in females; Trail Making Test-A shows right-eye dominance. Findings in Trail-Making-Test-A projected to left brain hemisphere, and the ratio incongruent to neutral in the Stroop test projected to right brain-hemisphere. Separate assessment for sex and eye-side is presented for the first time in a population-based study. Location-specific sectorial retinal nerve fibre layer thickness was found to be an indicator for cognitive performance, giving an option for early detection of cognitive decline and the potential of early treatment. The eye as a window to the brain was studied with optical coherence tomography and connected to cognition. Girbardt et al. report that thinner retinal nerve fibre layer thickness was found to be a meaningful index for poorer cognitive performance which presents the potential for prediction of future cognitive decline.

## Teaser

The eye as window to the brain was studied with optical coherence tomography and connected to cognition. Girbardt et al. report that thinner retinal nerve fiber layer thickness was found to be a meaningful index for poorer cognitive performance which presents the potential for prediction of future cognitive decline.

## Graphical Abstract

**Graphical Abstract:**
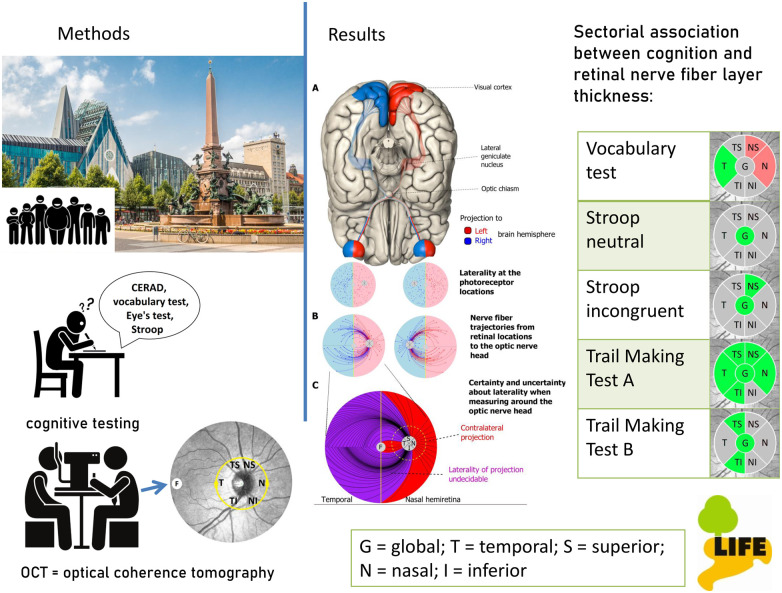

## Introduction

To measure cognitive abilities, extensive tests and test batteries have been developed. Modern imaging techniques are used in cognitive neuroscience to examine the neuroanatomical and functional correlates of cognitive abilities such as attention, language or memory.^1^^,^^2^ In the eye, as ‘window to the brain’, it is possible to examine early degeneration of the central nervous system fast and non-invasively at micrometre resolution using optical coherence tomography (OCT). As all retinal nerve fibres throughout the eye project to the optic nerve head, the circumpapillary area is the most informative region to detect nerve fibre damage in the eye. Retinal nerve fibre layer thickness (RNFLT) is an important measure to diagnosing optic neuropathies like glaucoma and has been shown to decrease with age in healthy individuals.^3–6^ Furthermore, there is evidence that RNFLT decreases with poorer cognitive abilities.^7^^,^^8^ Over 46 million people around the world live with dementia.^9^ As therapeutic options are very limited so far, there is a need to screen for the disease in an early stage to be able to develop new forms of treatment. An inexpensive and non-invasive marker like RNFLT which also serves other diagnostic purposes would be most suitable.

Previous population-based studies provide coarse results on retinal layers and cognition. Investigation of ‘macular’ ganglion cell inner plexiform layer in the large-scale ‘Rotterdam study’ (*n* = 3289) showed association with prevalent dementia.^10^ Less macular ganglion cell layer volume was associated with worse processing speed and worse verbal episodic memory in the ‘Rhineland study’ (*n* = 2483).^11^

Investigation of circumpapillary retinal nerve fibre layer thickness (cpRNFLT) in the Rotterdam study revealed association of thinner global values with low performance in several cognitive tests, this was supported by a trend in the Rhineland study.^10^^,^^11^

The ‘Three-City-Alienor study’ (*n* = 483) did not observe a significant association between cognitive performance and global RNFLT in baseline measurement, but sectorial analysis in follow-up showed a significant association of decreased RNFLT with memory decline (temporal, temporal-superior, temporal-inferior, nasal).^12^ The inferior area of RNFL was significantly related to general processing speed in the ‘Lothian Birth Cohort 1936’ (*n* = 96) in subjects aged 72 years.^13^ These two studies motivated this study, in which we investigated sectorial RNFLT in a much larger sample.

Other population-based studies without OCT technology have estimated RNFLT with scanning laser polarimetry and *in* *vivo* confocal microscopy (Heidelberg retinal tomography, HRT). ‘Erasmus Rucphen Family study’ (*n* = 1485) and ‘EPIC Norfolk Cohort study’ (*n* = 5563) found a positive association of global RNFLT with cognitive performance in several tests.^14^^,^^15^ An association between thinner global RNFLT and self-reported Alzheimer’s disease was found in the ‘Northern Ireland Cohort for the Longitudinal Study of Ageing’ (NICOLA) (*n* = 3221).^16^

Analyses on averaged macular RNFLT with the ‘UK Biobank data’ (*n* = 32  038) indicate ‘sector-specific’ rather than global relations when comparing with circumpapillary data. As macular nerve fibres project towards the optic nerve head via the inferior-temporal and temporal segments, these two segments can be compared to our data.^17^ These results motivated our study to specifically analyse not only the inferior-temporal and temporal segment but also ‘all’ segments around the optic nerve head.

Here, we aim to investigate RNFLT and cognitive performance in a large population-based sample by presenting age-, sex-, education- and scan radius-adjusted analyses separately for male and female subjects. The latter is important due to sex-dependent differences in RNFLT^18^ and cognition.^19^ While most previous studies summarized RNFLT globally or at most divided into coarse segments, we analysed RNFLT at greater precision of ‘six segments’. Furthermore, we consider effects of ocular laterality motivated by projections into different brain hemispheres for the first time in a population-based study.

We hypothesize that RNFLT can provide diagnostic information on cognitive decline. We specifically hypothesize thinner RNFLT to be associated with lower cognitive performance in a sector-specific manner. Additionally to previous studies focusing especially on Alzheimer’s disease (AD), we consider the entire spectrum of cognitive decline and focus on different neuropsychological tests in particular. Unlike case–control studies, our population-based approach is ideal to obtain a broad and sizable sample of individuals and to adjust for potential confounders.

## Materials and methods

### Study population

Participants were investigated in the framework of a large population-based cohort study conducted by the Leipzig Research Centre for Civilization Diseases (LIFE-Adult Study) at Leipzig University, from August 2011 to November 2014. LIFE-Adult includes 10 000 randomly selected participants from the population registry of over half a million inhabitants of Leipzig, Germany. Subjects were recruited in an age and gender stratified manner, focusing on the age range 40–80 years. More specifically, 9600 subjects between 40 and <80 years of age and 400 subjects between 19 and <40 years of age were recruited. All participants provided written informed consent prior to participation. The institutional ethics board of the Medical Faculty of Leipzig University approved the study and the research followed the Declaration of Helsinki.

**Figure 1:**
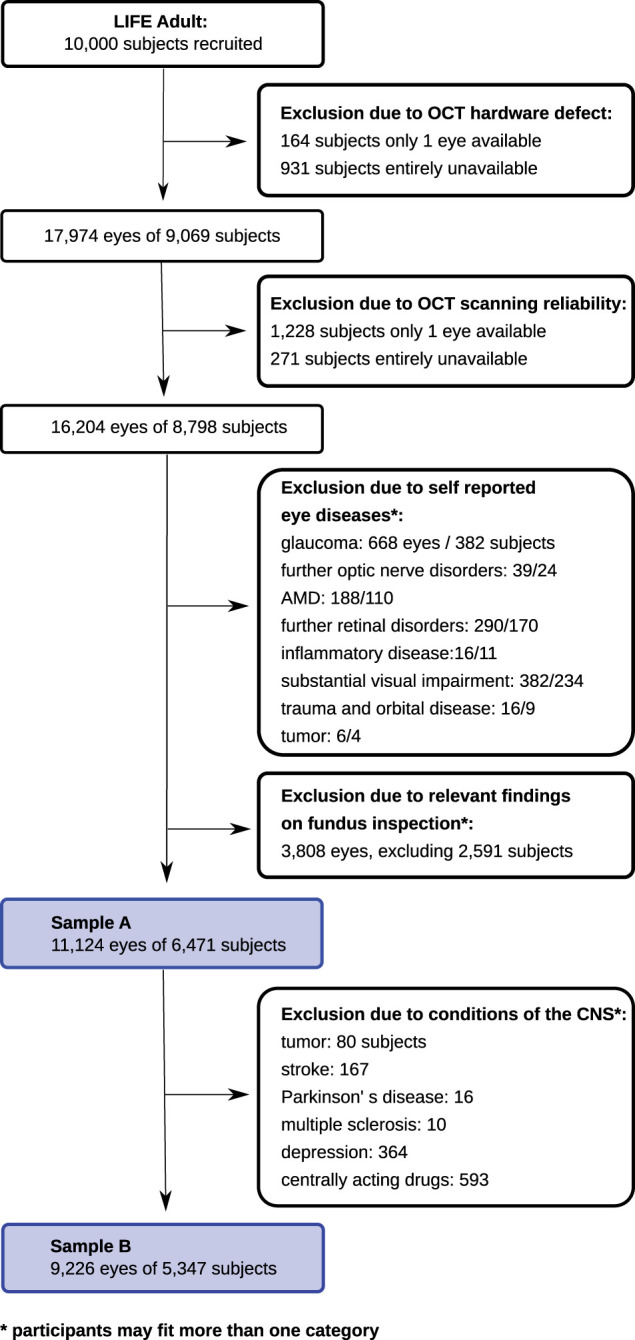
**Flowchart.** This flowchart depicts the exclusion algorithm. After exclusion of data based on image quality and ocular conditions, sample A consists of 11 124 eyes of 6471 subjects. To account for central confounders, we additionally excluded the following conditions: tumour, stroke, Parkinson’s disease, multiple sclerosis, epilepsy, depression and centrally acting drugs. This data set is labelled sample B, including 9226 eyes of 5347 subjects.

Of the 10 000 LIFE subjects, 9069 had cpRNFLT available. Of those, 271 subjects were excluded from the analysis sample because of unreliable OCT scanning quality. Another 2327 subjects were excluded due to relevant ophthalmic findings classified either based on current ophthalmological standards and/or medical history (i.e. optic nerve disorders including glaucoma, retinal disorders including age-related macular degeneration (AMD), inflammatory disease, substantial visual impairment, severe ocular trauma, orbital disease and tumours of the eye). See also Fig. 1 and Supplementary material 1 ‘exclusion criteria’ for more details.

We present two analyses. In the first sample A, we included all subjects based on exclusion criteria described above (11 124 eyes of 6471 subjects). Considering that several neurological or psychiatric conditions are known to affect brain function, and potentially RNFLT, we additionally excluded the following conditions: tumour, stroke, Parkinson’s disease, multiple sclerosis, epilepsy, depression and centrally acting drugs. This data set is labelled sample B, including 9226 eyes of 5347 subjects.

The final numbers of included subjects per cognitive assessment are depicted in Table 1 ‘characteristics of the study population’. The number of subjects varies between tests because some cognitive tests were performed on subsets of the LIFE participants.

**Table 1:** Characteristics of the study population

	Eyes, no.	Subjects, no.	Age, mean (2.5%, 97.5%)	Male, no. (%)	Right eyes, no. (%)	Test score (2.5%, 97.5%)
**Sample A**							

Entire sample A^a^	11 124	6471	55.5 (28.6, 76.3)	46.9	50.3	NA	
Boston Naming Test^a^	2169	1323	69.1 (60.7, 78.3)	52.3	50.3	14.5 (12, 15)	
MMSE^a^	2160	1318	69.1 (60.7, 78.3)	52.5	50.2	28.2 (24, 30)
MWT-B^a^	10 996	6392	55.4 (28.5, 76.3)	46.9	50.3	104.8 (72, 129)
Phonemic fluency ‘S’^a^	2171	1324	69.1 (60.7, 78.3)	52.3	50.3	12.8 (5, 22)
RMET^a^	2362	1378	56.6 (23.4, 77.1)	55.3	50.4	23.1 (15, 30)
Semantic fluency^a^	11 091	6450	55.5 (28.6, 76.3)	46.9	50.3	24.2 (13.0, 38.0)
Stroop neutral^b^	10 667	6196	55.3 (28.5, 76.3)	46.7	50.3	1669 (1065, 2704)
Stroop inc.^b^	10 667	6196	55.3 (28.5, 76.3)	46.7	50.3	1975 (1137, 3341)
Stroop inc./neut.	10 671	6198	55.3 (28.5, 76.3)	46.7	50.3	1.2 (0.9, 1.5)
TMT-A^b^	11 093	6453	55.5 (28.6, 76.3)	46.9	50.3	35.2 (17, 70)
TMT-B^b^	11 024	6413	55.4 (28.5, 76.3)	46.8	50.3	83.7 (38, 209)
TMT B/A	11 016	6408	55.4 (28.5, 76.3)	46.8	50.3	2.4 (1.3, 4.8)
Visuoconstruction copy^a^	2161	1318	69.1 (60.7, 78.3)	52.4	50.3	10.7 (8, 11)
Visuoconstruction recall^a^	2142	1306	69.1 (60.7, 78.3)	52.5	50.4	10.8 (5, 14)
Wordlist learning^a^	3369	1993	59.6 (24.1, 77.1)	52.2	50.3	22.6 (14, 29)
Wordlist recall^a^	3369	1993	59.6 (24.1, 77.7)	52.2	50.3	8.0 (4, 10)
Wordlist recognition^a^	3357	1985	59.6 (24.2, 77.7)	52.1	50.4	19.7 (18, 20)

**Sample B**							
Entire sample B	9226	5347	54.8 (27.7, 76.0)	49	50.2	NA
Boston Naming Test^a^	1679	1020	68.9 (60.6, 78.3)	55.2	50.7	14.5 (12, 15)
MMSE^a^	1672	1016	69.0 (60.7, 78.3)	55.4	50.7	28.3 (25, 30)
MWT-B^a^	9126	5285	54.7 (27.7, 75.9)	49.0	50.2	105.4 (75, 129)
Phonemic fluency ‘S’^a^	1681	1021	68.9 (60.6, 78.3)	55.1	50.7	12.9 (5, 22)
RMET^a^	1922	1119	55.5 (23.3, 76.9)	56.8	50.7	23.2 (15, 30)
Semantic fluency^a^	9199	5330	54.8 (27.7, 76.0)	49.0	50.2	24.4 (13.0, 38)
Stroop neutral^b^	8863	5129	54.6 (27.7, 75.9)	48.9	50.3	1649 (1062, 2651)
Stroop inc.^b^	8861	5128	54.6 (27.7, 75.9)	48.9	50.3	1954 (1136, 3305)
Stroop inc./neut.	8863	5129	54.6 (27.7, 75.9)	48.9	50.3	1.2 (0.9, 1.5)
TMT-A^b^	9200	5332	54.8 (27.7, 76.0)	49.0	50.2	34.2 (17, 67)
TMT-B^b^	9155	5306	54.7 (27.7, 76.0)	49.0	50.2	81.1 (38, 198)
TMT B/A	9147	5301	54.7 (27.7, 76.0)	49.0	50.2	2.4 (1.3, 4.7)
Visuoconstruction copy^a^	1673	1016	69.0 (60.7, 78.3)	55.3	50.7	10.7 (8, 11)
Visuoconstruction recall^a^	1661	1009	68.9 (60.7, 78.3)	55.5	50.8	10.9 (5, 14)
Wordlist learning^a^	2719	1604	58.6 (23.8, 77.5)	54.2	50.6	22.8 (15, 29)
Wordlist recall^a^	2719	1604	58.6 (23.8, 77.5)	54.2	50.6	8.1 (4, 10)
Wordlist recognition^a^	2707	1596	58.6 (24.0, 77.6)	54.1	50.6	19.7 (18, 20)

inc. = incongruent; neut. = neutral; MMSE = Mini-Mental state examination; NA = not applicable; RMET = Reading the Mind in the Eyes test; TMT = Trail Making Test.

a Score in points.

b Time in seconds.

### Ophthalmologic assessments

Ophthalmological imaging included spectral-domain OCT (Spectralis, Heidelberg Engineering, Heidelberg, Germany, Spectralis HRA + OCT, Acquisition Module 5.4.7.0). OCT was performed to obtain cpRNFLT scans with a resolution of 768 equidistant measurement points placed on a circle around the optic nerve head. The circle location and its coordinate system are illustrated by Wang et al. RNFLT are evaluated in six segments (T: temporal, TS: temporal-superior, TI: temporal-inferior, N: nasal, NS: nasal-superior, NI: nasal-inferior) and overall average (G). These sectorial data are analysed in comparison with cognitive assessments for the current analysis.

We included both eyes per subject wherever possible. We have previously described for the same dataset in a high level of detail the similarities and differences between right and left eyes within the same person.^20^ Given the existing interocular symmetries on subject-specific level, a mixed linear regression model with random effects for subjects was considered but proved to be too complex given the large number of different subjects as a ratio of the total number of samples (each subject had at most two measurement samples: right and left eye). Being aware of this issue, we performed separate data analyses for right and left eyes to explicitly investigate potential ocular laterality effects on RNFLT. Reliability criteria for the OCT scan were based on: (i) image quality with signal-to-noise-ratio ≥20 dB; (ii) average number of B-scans ≥50; and (iii) no more than 5% missing or unreliable cpRNFLT segmentations among the 768 A-scans which are the basis of the average sectorial data. All evaluated scans were adequately centred on the optic nerve head.

Previous studies have demonstrated strong relationships between RNFLT and age^21^ and sex.^18^ In addition, it has been shown that ocular magnification effects, due to corneal curvature, lens-related myopia or axial length, have a substantial impact on the true size of the OCT scanning circle.^21^ The Spectralis machine estimates the true scan radius from the individual focus settings of each scan. We used this estimated true scan radius as an additional covariate.

### Neuropsychological Assessment

A detailed description of the performance and intention concerning the following cognitive assessments are described in Supplementary material 2 ‘neuropsychological assessment’. Note that the individual cognitive phenotyping included various cognitive dimensions, including memory, attention, executive and language functions, social cognition and others. We used the authorized German version of the CERAD Plus test battery (The Consortium to Establish a Registry for Alzheimer’s Disease), an established battery for phenotyping AD and related disorders, which included the Mini Mental State Examination (MMSE), Boston Naming Test (BNT), Phonemic fluency, Semantic fluency animals, Trail Making Tests (TMT), Visuoconstruction copy, Visuoconstruction delayed recall, Wordlist learning, Wordlist delayed recall and Wordlist recognition.^22^^,^^23^ Further tests were the Reading the Mind in the Eyes test (RMET),^24^ Multiple choice German vocabulary test (MWT-B)^25^ and the Stroop-Test.^26^^,^^27^ Education is known to have an influence on cognitive testing,^28^^,^^29^ accordingly, we included education as a covariate in our analyses.

### Statistical analysis

We analysed associations between RNFLT, separately for each of the six segments as well as globally, and performance in the several cognitive tests in two different ways: First, by separate Pearson’s correlations with each cognitive score, and second, by multivariable linear regression with RNFLT as outcome and each cognitive test score as well as age, the estimated true scan radius, sex and education as regressors: RNFLT = β_0_ + β_1_ (cognitive test) + β_2_ (age)+ β_3_ (sex) + β_4_ (scan radius)+ β_5_ (education). We investigated males and females separately in additional sub-analyses. Moreover, the data analyses were performed separately for right and left eyes to investigate possible relationships with brain laterality, as explained below. Significant relationships (*P* < 0.05) were determined after adjusting the sectorial and global results for multiple comparisons by false discovery rate. All statistical analyses were performed in R environment using version 3.6 (R Foundation for Statistical Computing, Vienna, Austria).

### Brain hemisphere-specific correlation by evaluating nasal, nasal superior and temporal cpRNFL

**Figure 2:**
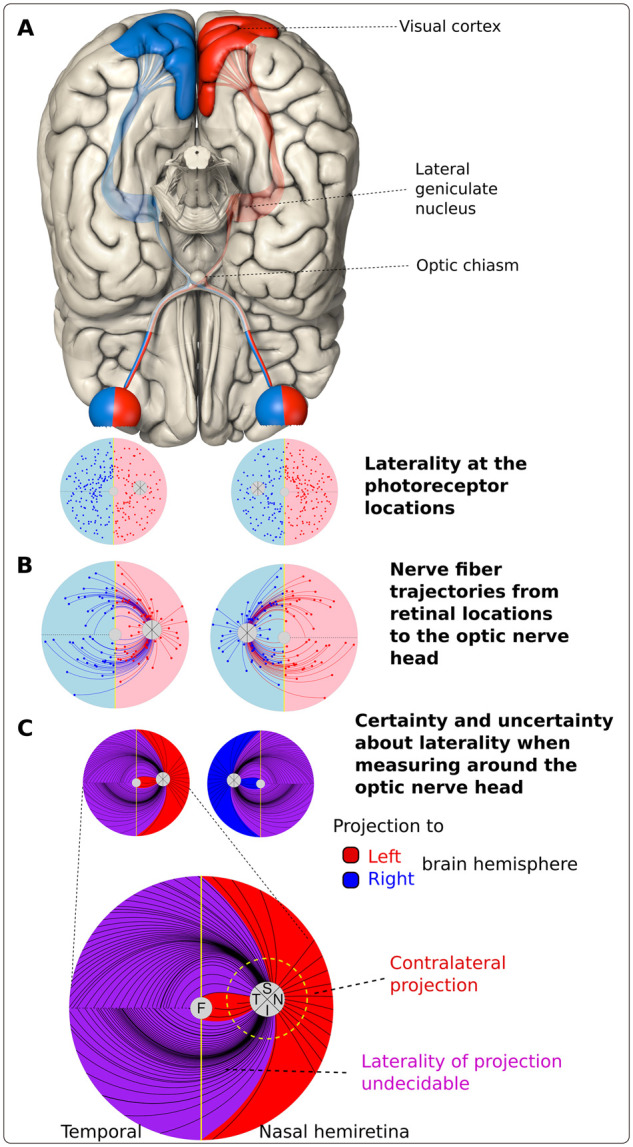
**Eye to brain projection.** Illustration of the retinal nerve fibre projections from the hemiretinae to the corresponding brain hemispheres. A vertical meridian divides the retina at the fovea into two parts. The information of the left hemiretinae is processed in the left visual cortex because axons of the nasal retinal ganglion cells cross in the optic chiasm to the contralateral brain-side and temporal originating fibres stay ipsilateral. This is identical for the right hemiretinae (**A**). Nerve fibre trajectories within the eye projecting to the optic nerve head (ONH), here illustrated according to the Jansonius model,^30^ can traverse the vertical midline (**B**). Therefore, when measuring around the ONH, the fibres passing certain circumpapillary sectors project both to the right and to the left brain hemisphere and can therefore not be definitely assigned to a specific brain hemisphere (**C**). This perspective differs from the standard illustration (**A**), which refers to the retinal ganglion cell bodies and retinal layers underneath at each retinal location. In **C**, we marked retinal areas where only crossing fibres are located in red for the right eye and in blue for the left eye (T, S: superior, N, I: inferior circumpapillary sector). Purple marked zones contain superimposed fibres from both hemiretinae, which cannot be allocated to one specific brain hemisphere from the location of our measurement.

Retinal nerve fibres originating from the macular bundle proceed to the temporal peripapillary segment. These and the peripapillary nasal and nasal superior located fibres decussate to the contralateral brain hemisphere. Therefore, we correlated the measurements of these three segments to one brain hemisphere. Regarding the remaining peripapillary segments, direct relationship with one brain hemisphere is not possible because in these segments, axons from the superior and inferior nasal ganglion cells as well as the arcuating temporal axons are intermingled, see Fig. 2 for details.

### Data availability

The authors confirm that the data supporting the findings of this study are available within the article and its Supplementary materials. All presented data are available as Supplementary information for download. Raw data cannot be shared publicly because of consent restrictions of LIFE-Adult participants. Data are available after an approved project agreement from the LIFE Leipzig Research Centre for Civilization Diseases. Please contact Dr Matthias Nüchter (Head of LIFE Managing Office, contact via matthias.nuechter[at]life.uni-leipzig.de) for data access requests.

## Results

### Demographic characteristics

The final analysis included 11 124 eyes from 6471 participants (mean age 55.5 years, range 19.1–79.8 years, 46.86% male). Table 1 shows the number of subjects per test and demographic characteristics. The study population is almost exclusively of European descent (0.86% in sample A and 1.2% in sample B were non-Europeans). Education was classified on the basis of the CASMIN-scale (Comparative Analysis of Social Mobility in Industrial Nations)^31^: 7.24% in sample A and 6.47% in sample B had lower education, 58.64% in A and 58.25% in B had intermediate education and 34.12% in A and 35.28% in B had high education.

Age was found to be inversely related to performance in the following cognitive measures: MMSE, Wordlist learning, recall, recognition, RMET, Semantic fluency, TMT, Stroop neutral-, and -incongruent. Mean RNFLT was 97.3 µm (SD 9.3 µm) and 97.4 µm (SD 9.3 µm) for sample A and sample B; see Supplementary material 3 ‘Results’ for details.

### Association between RNFLT and performance in cognitive tests

**Figure 3:**
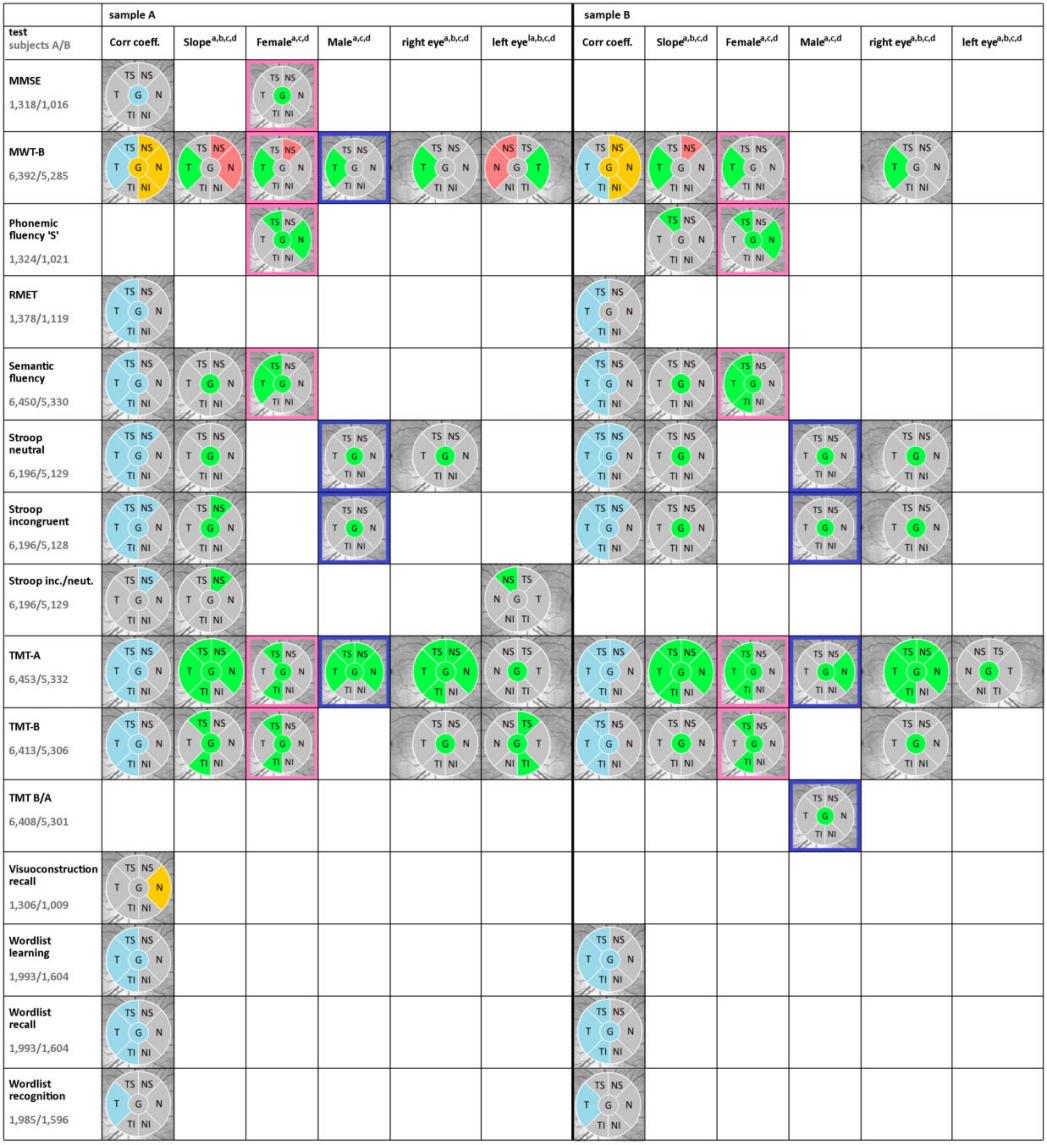
**Associations of sectorial cpRNFLT with cognitive tests.** Figure  3 shows statistically significant associations of RNFLT in six segments and overall average with performance of neuropsychological tests for sample A (without exclusion of central confounders) and sample B (with exclusion of several conditions affecting the central nervous system). All results are pictured as right eyes except for the separate analysis of left eyes. Thicker or, conversely, thinner RNFL in association with better cognitive test results is marked in blue respectively yellow for correlation analysis and in green respectively red for regression analysis with adjustment for age, sex, scan radius and education. The first column depicts the correlation without any adjustment. The second column presents our data with adjustment for age, sex, scan radius, and education. The next two columns present the data separated for female and male subjects and finally we show an analysis for right and left eyes of our sample. No statistically significant correlation of cognition and RNFLT was found for Boston Naming Test, and Figure Copy which is not depicted. All numerical details can be found in Supplementary material 3 ‘Results’. One example on how to read the figure: Good performance in the CERAD test semantic fluency correlated statistically significant with thicker RNFLT in the segments G, T, TS, TI in sample A and B. After multivariable regression analysis with adjustment for age, sex, scan radius, and education, thicker global RNFLT remained statistically significant in both samples. Separate analysis for females and males with adjustment for age, scan radius, and education had statistically significant regression only in females in the segments G, T, TS in sample A and in the segments G, T, TS, TI in sample B. Separate analysis for right and left eyes with adjustment for age, sex, scan radius and education revealed no statistically significant regression in both samples. Adjustment for age^a^, sex^b^, scan radius^c^, education^d^. Corr. coeff. = correlation coefficient; G = global, overall average; inc. = incongruent; MMSE = Mini-Mental State Examination; MWT-B = Multiple choice German vocabulary test; N = nasal; neut. = neutral; NI = nasal-inferior; NS = nasal-superior; RMET = Reading the Mind in the Eyes Test; slope = regression coefficient; T = temporal; TI = temporal-inferior; TMT = Trail Making Test; TS = temporal-superior.

Figure 3 presents RNFLT in six segments (T: temporal; TS: temporal-superior; TI: temporal-inferior; N: nasal; NS: nasal-superior; NI: nasal-inferior, G: global, overall average) and its statistically significant associations with cognitive tests. Numerical results are shown in Supplementary material 3 ‘Results’.

In the majority of the implemented tests, better performance was associated with thicker RNFLT, while lower scores were related to thinner RNFLT, respectively. MWT-B and Figure Recall show associations of better test performance with thinner RNFL in predominantly nasally located segments. RNFLT was not associated with performance in BNT and Visuoconstruction copy.

Sample A and B present similar results in all six analyses. In sample B, additional significant segments were found in Phonemic fluency (slope TS), Semantic fluency (females TI), TMT-A (females T), TMT B/A (males G) Stroop incongruent (right eye G). Correlation analysis in both samples revealed predominant associations globally and in temporally located segments.

After multivariable regression analysis with adjustment for age, sex, scan radius and education, five tests still show statistically significant positive associations globally in both samples. In this analysis, the previously predominant associations in temporally located segments are considerably reduced. The maximum of segments with statistically significant association is found for TMT-A in both samples (G, T, TS, TI, N and NS).

### Sex-specific analyses

Separate analyses for females and males, see Fig. 3, demonstrate that, in both samples, some associations occur predominantly in one sex and other associations are distributed asymmetrically. Female-only associations (positive) were found in semantic fluency, MMSE, TMT-B and phonemic fluency, mainly concerning sectors G and TS. In men, associations (positive) were solely found globally in Stroop neutral and incongruent condition and ratio TMT B/A.

### Eye laterality

Five cognitive tests investigated have asymmetrical results between right and left eyes, see Fig. 3. For the right eye, associated segments of samples A and B are identical.

Noticeably, in sample B, except for TMT-A, all statistically significant associations concern the right eye.

### Brain hemisphere-specific analysis

In our separate analysis of unilateral associations of segments N, NS and T, where fibres mainly cross to the contralateral brain hemisphere (see Fig. 2), when adjusting for sex, age and scan radius only, Semantic fluency and TMT-A in both samples, and TMT-B in sample A were positively associated with RNFLT of the right eye, projecting to the left hemisphere. Ratio Stroop incongruent/neutral was negatively associated with the left eye in sample A. After additional adjustment for education, the mentioned associations of TMT-A and ratio Stroop incongruent/neutral were still present. MWT-B in sample A had positive temporal association in both eyes with RNFLT but additional negative association in N and NS for the left eye.

We found associations of better cognitive test performance with both thicker RNFLT in some and thinner RNFLT in other segments only for the MWT-B, where thickening was located temporally and thinning in segments N and NS.

Interestingly, the segment NI shows the least effects apart from MWT-B in both samples.

**Figure 4:**
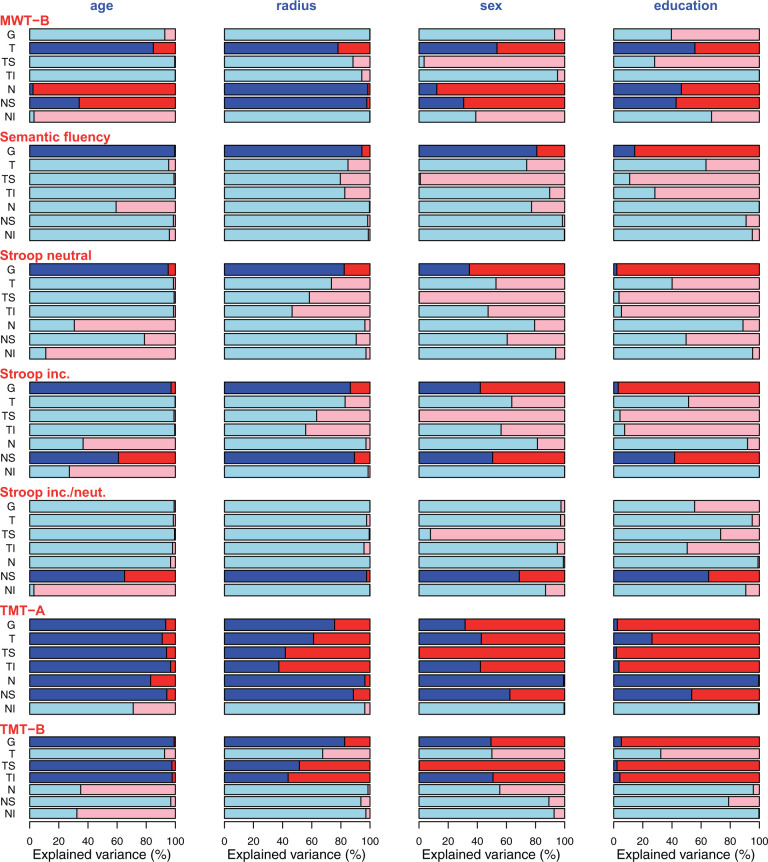
**Ratio of variance sample A.** Sample A: Ratio of variance explained by the respective cognitive test score (red) that is not explained by each covariate (blue; from left to right: age, radius, sex, education) relative to the total explained variance. Only tests with significant slopes were included. Segments for which the cognitive test score was not significant after correcting for the covariates are shown in pale colours. In other words, the red part of each bar illustrates the percentage of variance that is explained in addition to the variance that is already explained by age, radius, sex, education, respectively.

**Figure 5:**
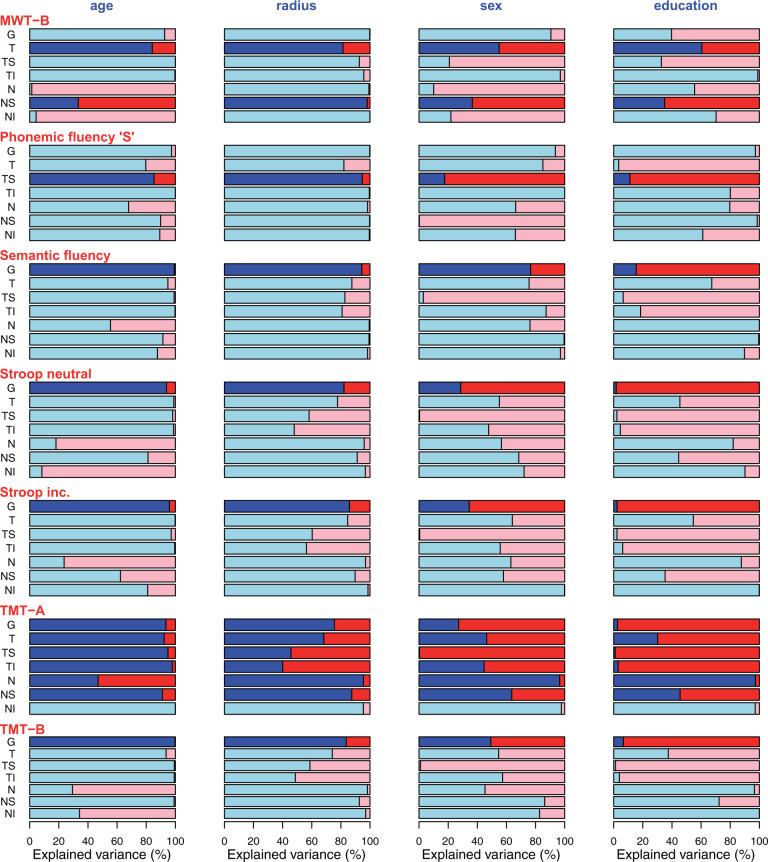
**Ratio of variance sample B.** Sample B: Ratio of variance explained by the respective cognitive test score (red) that is not explained by each covariate (blue; from left to right: age, radius, sex, education) relative to the total explained variance. Only tests with significant slopes were included. Segments for which the cognitive test score was not significant after correcting for the covariates are shown in pale colours. In other words, the red part of each bar illustrates the percentage of variance that is explained in addition to the variance that is already explained by age, radius, sex, education, respectively.

Additionally explained variance by cognitive test scores (effect size). As detailed elsewhere in this work, age, scan radius and sex have previously been shown to have substantial impacts on RNFLT and, therefore, explain considerable amounts of RNFLT variance. Furthermore, education as a parameter is known to be correlated with the scores of many cognitive tests. Associations between RNFLT and cognitive test scores become particularly relevant if the test score explains any additional variance which is not already explained by these available covariates. To quantify this additionally explained variance, we first cumulatively calculated the entire variance explained by the four parameters age, scan radius, sex and education and then determined the remaining explained variance, which can then solely be attributed to the test score. Figures 4 and 5 show the ratios of variance explained by each respective cognitive test score (red) that is not explained by the covariates (blue) age, radius, sex and education relative to the total explained variance for sample A (Fig. 4) and sample B (Fig. 5). In other words, these figures illustrate how much additional variance each cognitive test explains for each sector and each covariate.

## Discussion

In this large population-based cohort study, we found significant statistical association of poor cognitive performance in several neuropsychological tests predominantly with thinner cpRNFLT. Mean RNFLT (see Supplementary material 3 ‘Results’) is in line with normative data of European and Asian populations.^32^^,^^33^ We previously found an annual decrease of RNFLT of 0.17 µm when adjusting for scan radius.^21^ We further demonstrated both sex-specific differences^18^ as well as differences in laterality^20^ of RNFLT. Comparable to other data, cognitive performance for most tests was lower with higher age.^34^

As detailed in the Results and further discussed below, in this study, we deliberately analysed associations between RNFLT and cognitive performance at an unprecedented level of detail, which resulted in a large number of different conditions together with their respective subgroups and thereby in a multitude of statistical comparisons. Some of the effects robustly appear under multiple conditions, such as the association of RNFLT with attention (TMT) or the focus on retinal locations in superior sectors. Other findings are restricted to only single conditions or subgroups and are therefore more difficult to interpret. Nevertheless, these effects survived our adjustments for multiple comparisons, which is why we report them here as well. These seemingly isolated effects, many of which could not have been found in previous studies as the respective combination of conditions had not been investigated before, may inspire specifically designed future studies which could help to better understand and interpret them and reveal potential underlying conditions for them.

Effects, which occur only in sample B, indicate that our decision to exclude possible brain-related confounders was justified as it highlights associations with RNFLT despite the smaller sample size.

In many neuropsychological tests, we found effects in temporal segments (T, TS and TI), which were considerably reduced when adjusting for age, sex, scan radius and education. Similarly, Feuer et al.^35^ found the biggest thinning per decade in segments NS, TS, N, TI for their combined cohorts and Parikh et al.^36^ found the main thinning per decade in segments NS, TS, TI, adapted from publication of 12 segments. As we previously demonstrated, thinning of RNFLT is underestimated in higher ages when not adjusting for scan radius.^21^

We found remarkable sex differences. Female eyes exclusively presented effects in MMSE, Fluency tasks and TMT-B in the dominant segments G, TS and TI. Only in men, Stroop neutral and incongruent had global effects. As we recently published regarding the LIFE cohort, global RNFLT is 1 µm larger in females with location-specific differences up to 9.98 µm nasally. All over, we found areas with thicker RNFLT in females within the segments T, TS, TI, N and NI and within segments ST and NS for males.^18^ Analogously, in the Gobi Desert Children Eye study, girls had thicker RNFL than boys in segments T, TI, N, NS.^37^ In addition, females of the LIFE cohort performed better than men in many cognitive tests besides BNT and Constructional praxis.^19^ Differences between females and males as observed in our study might be related to these factors, that is, sex-specific differences in RNFLT and cognition.^18^^,^^37^ Accordingly, separate analysis of female and male eyes is generally advisable in future studies, matching a recent focus to consider sex-specific analyses in neuroscience in general.^38–41^ Previous population studies included sex as covariate but did not publish analyses for single tests stratified by sex.

Concerning eye laterality, we found effects in sample B almost only for the right eye. In TMT-A, for the right eye in both samples, we found significant effects in 6 of 7 segments whereas in the left eye only the global mean is significant. Global RNFLT is slightly thicker in right eyes. As we previously showed, the thickness profile of cpRNFL is location-specific with thicker areas for right eyes in segment N and thicker areas for left eyes in segment NS.^20^ To our knowledge, the impact of cognition on RNFLT with differentiation of eye laterality has not been demonstrated in a population-based study before.

Most fibres of the segments N, NS and T cross to the contralateral brain hemisphere. Analogously, in patients with posterior cerebral artery infarction, the lesions in RNFL due to transsynaptic retrograde degeneration were least in segments N and NS of ipsilateral eyes while contralateral thinning was found in all segments.^42^ This indicates that performance of TMT-A might correlate particularly with changes in the left brain hemisphere. This finding is in line with results from lesion studies which provide evidence for association of TMT with several locations within the left frontal and parietal lobe.^43^^,^^44^ TMT-B correlated only with RNFLT when not adjusting for education. The ratio Stroop incongruent/neutral correlated in segment NS of the left eye from where fibres are connected with the right brain hemisphere. Although this finding in a verbal interference task might contradict the fMRI and brain lesion model literature at first glance,^45^ several studies reported also bilateral brain activations^46^ strengthened by a recent lesion model.^47^ The Stroop task is related to bilateral frontal region and the core network for tasks requiring supervisory control for the suppression of a routine action in favour of another, non-routine action was even related specifically to the right anterior insula and inferior frontal junctions.^48^

The MWT-B which estimates crystallized intelligence is the only test where better results were associated with thicker RNFLT in temporal segments and simultaneously with thinner RNFLT in nasal segments. Accordingly, global RNFL showed no association in 10 of 12 subanalyses because the effects were lost due to averaging. For this reason, it is important to analyse detailed segments. For Visuoconstruction recall, we also found opposite effects in correlation of better test results with nasal thinning. An explanation for these needs to be investigated in further studies. Few case-control studies found an augmentation of RNFLT in patients with mild cognitive impairment compared to healthy adults and discussed this as a result of gliosis which precedes axonal degeneration and tissue thinning.^49^^,^^50^

Even though the ratios of variance explained in addition to the covariates (Figures 4 and 5) show that the global RNFLT is in many cases almost fully explained by age, at many of the single separate RNFLT segments, more variance is explained by the cognitive test versus age. This underlines the benefit of analysing cpRNFLT at a higher spatial resolution compared to the global average. Another noteworthy conclusion from these figures is the large additional variance explanation of the cognitive tests compared to education.

Several previous population-based studies have investigated the impact of cognition on RNFLT, focusing on different aspects. Five studies present data for global RNFLT alone.^10^^,^^11^^,^^13–15^ The Lothian Birth cohort^13^ gives results for four segments. Only the Three-City-Alienor study^12^ provides a spatial resolution of six segments as our study, however, for much less participants and fewer cognitive tests. The UK Biobank^17^ analyses macular RNFL which can be matched to our segments T, TI, in the Erasmus Rucphen study,^14^ scanning laser polarimetry is used and in the Epic Norfolk Cohort,^15^ RNFL is derived with HRT. Those are different technologies and their results might not be easily comparable to the OCT-derived data of the other studies.

‘TMT’ as common assessment for attention, cognitive speed (TMT-A), and flexibility (TMT-B) is frequently impaired in brain disorders and, therefore, our outstanding associations of this test with RNFLT give meaningful evidence for OCT-investigation for cognitive screening. Additionally to cognitive challenge, TMT is also visually demanding.

For ‘TMT-A’, other population-based studies also found an association of thinner RNFLT with worse results for cognitive performance: Rotterdam Study^10^ globally using letter-digit substitution test, UK-Biobank^17^ for segments T, TI with timed test of symbol matching (macular RNFLT). In the Epic Norfolk study,^51^ the related letter-cancellation test was associated with RNFLT measured with HRT but only when not fully adjusting; in the Lothian Birth Cohort,^13^ general processing speed was associated with the inferior quadrant, and in the Rhineland Study,^11^ a statistically non-significant trend in TMT-A was found.

Concerning ‘TMT-B’, in the Erasmus Rucphen Family study,^14^ better performance was associated with thicker global RNFLT, as in our study. Astonishingly, this concerned predominantly younger individuals in their study. The Rotterdam Study^10^ showed association of RNFLT with letter-digit substitution test and in the Rhineland Study,^11^ a positive trend for TMT-B was found.

‘MMSE’, as general cognitive screening test, had only effects in global RNFLT in our analysis of females in sample A. The test has a ceiling effect as the majority of our participants had no dementia. In the Rotterdam Study,^10^ a positive association was only found for baseline measurements and the Epic Norfolk Study^15^ had a positive association of HRT-derived RNFLT with the short-form MMSE. In the Lothian Birth Cohort,^13^ general cognitive ability was negatively associated with RNFLT, however, this was only the case when including former IQ at age 11 as covariate. They discussed the relatively good cognitive health of their cohort as possible reason for this converse result. However, our population sample with a mean MMSE of 28 (95% CI: 24–30) was also in good cognitive health.

As verbal fluency can be impaired very early in AD,^52^ these test results, relating to several processes for example language and flexibility are of particular interest. We found global association for ‘semantic fluency’^53^ in both samples, analogous to the Erasmus Rucphen Family Study.^14^ Rotterdam Study,^10^ Three-City-Alienor Study^12^ and Epic Norfolk Study^15^ found no association of semantic fluency with RNFLT. In our analysis, ‘phonemic fluency’ had effects on RNFLT especially in females; other population-based studies did not present comparable tests.

As AD is the most prevalent type of dementia, we had expected to see an effect in ‘Delayed recall’; this might not be the case due to few cognitively impaired subjects. Memory tests in our analysis were only associated in correlation- but not in regression analysis. In the Erasmus Rucphen Family Study^14^ and the Epic Norfolk Cohort (HRT),^15^ memory tests were associated with global RNFLT, in UK Biobank^17^ in T and TI. In the Three-City-Alienor Study,^12^ RNFLT was not associated with memory at baseline. However, baseline RNFL was associated with lower decline in follow-up data for Free Delayed Recall (T, TS, TI) and Free plus Cued Delayed Recall (TI, N), both testing episodic memory. The Rhineland Study^11^ again found a trend, in the Rotterdam Study^10^ and Lothian Birth Cohort^13^ no association was found.

The ‘Stroop’ test is regarded as a measure of effectiveness of focused attention and additionally tests executive function, requiring inhibitory/interference control.^54^ We found global association in both samples likewise to the Rotterdam Study^10^ and the Erasmus Rucphen Family Study.^14^ Additionally, we found segment NS to be associated with sample A.

‘MWT-B’, differing from the other used tests, is a measure of crystallized intelligence. The Rhineland Study^11^ performed the identical test in which better performance was non-significantly associated with thicker RNFL but in the Epic Norfolk Study,^15^ where the short form of the National Adult Reading Test (NART) was evaluated, the results were statistically significant.

For the ‘Reading the Mind in the Eyes-Test’ as a measure of social cognition, we found no effects. To our knowledge, impact of this test on RNFLT was not investigated in a population-based setting before.

Summing up our results and those of other population-based studies, RNFLT might be useful as a biomarker for cognitive speed, executive functions and cognitive interference.

### Choice of RNFLT as an outcome

Most likely, both RNFL thinning and cognitive decline are indicators of general, systemic decline within the nervous system. Hence, correlation analyses between cognitive parameters and cpRNFLT first provided a quantification of this association regardless of any causal relationships. Second, multivariate regression analyses were carried out with a goal to establish if cpRNFLT, while correlated, would be indeed a useful variable to be associated with cognitive performance or if cognitive parameters would not add a significant amount of explained variance in addition to those parameters which were previously identified as the strongest impact factors on cpRNFLT. In our own previous studies on cpRNFLT, we identified age and scan radius influence cpRNFLT, with a particularly strong impact of scan radius (an eye-specific parameter, with cpRNFLT as outcome variable).^21^ Thus, we investigated if adding cognitive parameters to this set of previously identified strong covariates of cpRNFLT would explain any additional variance. Our rationale was: If cognitive parameters were to add any explained variance in addition to these known strong covariates, a typical ophthalmic OCT scan (which should include at least age and scan radius) has the potential to be associated with cognitive performance. In other words, we aimed to mimic a typical clinical ophthalmic measurement procedure, including standard covariates, and studied if this instrument could be sensibly related to cognitive parameters. Our design, therefore, investigates necessary conditions to associate cpRNFLT with cognitive decline.

To explicitly study cognitive decline, longitudinal studies are necessary. For this, the longitudinal course of cognitive test scores should indeed be the outcome variable and the choice of the covariates should not contain eye-specific parameters (e.g. scan radius) but rather a set of previously identified impacts on cognitive performance. Our cross-sectional dataset with eye-focused analyses provides the foundations to justify such longitudinal studies with cognitive performance over time as an outcome.

### Pathophysiological mechanisms of RNFLT alteration

A number of studies addressed RNFLT alteration by the influence of neurological diseases like stroke,^55^ multiple sclerosis^56^ or Parkinson’s disease.^57^ Causes for these might overlap with reasons for reduced RNFLT in cognitive decline: Changes of RNFL can concern all comprised components measured by OCT, for example, axons of the ganglion cells, glia cells, blood vessels.^58^ Damage can arise locally in the retina or originate more distantly as a consequence of axonal damage. Locally, deposits of amyloid beta plaques which are typically associated with AD, have been histologically found in the superior segment,^59^^,^^60^ where we also found significant effects. Damage in the optic tract can lead to thinning of RNFL as direct retrograde degeneration^61^ and cortical damage can even lead to transsynaptic retrograde degeneration.^62–64^ In a histological examination of the primary visual cortex in patients with AD, a greater density of senile plaques and neurofibrillary tangles was found in the cuneal gyrus (which corresponds to the superior retina) than in the lingual gyrus.^65^ This could explain the previously described inferior visual field defects in AD patients.^66^

As RNFLT is correlated with vessel density, it is also possible that RNFL thinning in our study is the result of vascular pathology. For instance, a study in patients with subcortical vascular cognitive impairment using OCT angiography found decreased temporal peripapillary capillary density.^67^

### Limitations

Subjects with severe cognitive or physical limitations might not have responded to an invitation to participate in the LIFE study. This might present a selection bias towards cognitively more healthy participants, however, this concerns all population-based studies. Our subjects were predominantly of European descent and our results are not necessarily generalizable.

As it is common practice in population-based studies on this topic, we excluded participants with glaucoma, since this optic neuropathy might confound the associations studied here. Therefore, our results are not fully implementable for glaucoma patients.

Most CERAD tests had a smaller sample size than other tests, which reduces the power of our data analyses. It might be possible that this reduced power contributed to fewer significant results for these tests.

While our results are promising in indicating RNFLT as an early marker for cognitive decline, our study is restricted to cross-sectional data and lacking longitudinal data to investigate this effect over time.

### Strengths

Among the strengths of our study is the large number of participants on which we performed extensive neurocognitive testing in a standardized setting. Furthermore, the high spatial resolution of six RNFL-segments enables us to present findings, which are not visible when analysing fewer sectors or the global mean alone. We clearly demonstrate the benefits of this high spatial resolution, and our results might motivate future studies with even higher sectorial resolutions as well as studies including larger retinal areas, such as a combination of peripapillary and macular volume scans at high spatial resolutions.

Moreover, to our knowledge, the impact of cognition on RNFLT with separate analysis of right and left eyes and furthermore distinguished analysis separated by sex has not been accounted for in a population-based study so far.

## Conclusions

We associate cpRNFLT using a spatial resolution of six segments with a considerable number of cognitive tests based on the largest population-based sample to date. We demonstrate highly location-specific effects of RNFLT segments with lower cognitive performance in several dimensions, particularly for trail making, stroop, and vocabulary tests. As common ophthalmic OCT devices analyse cpRNFLT in sector-specific ways, our findings are easily applicable in clinical practice. To summarize, our study not only confirms the previously reported general relationship between cognitive performance and RNFLT but also reveals the specific cpRNFLT regions that are most informative for each cognitive test. This improves the high potential for ocular OCT to be used as a possible adjunct or screening tool for detecting the onset of cognitive decline or monitoring its progression.

## Supplementary material

Supplementary material is available at *Brain Communications* online.

## Supplementary Material

fcab258_Supplementary_Data

## Abbreviations

AMD =: age-related macular degeneration
BNT =: Boston Naming Test
CERAD =: The Consortium to Establish a Registry for Alzheimer’s Disease
95% CI =: 95% confidence interval
cpRNFLT =: circumpapillary retinal nerve fibre layer thickness
dB =: decibel
G =: global
HRT =: Heidelberg retinal tomography
IQ =: intelligence quotient
LIFE =: Leipzig Research Centre for Civilization Diseases
MMSE =: mini mental state examination
MWT-B =: Multiple choice German vocabulary test
N =: nasal
NI =: nasal-inferior
NS =: nasal-superior
OCT =: optical coherence tomography
RMET =: Reading the Mind in the Eyes test
T =: temporal
TI =: temporal-inferior
TMT =: Trail Making Test
TS =: temporal-superior
