# Supplementary material for: Reading cognition from the eyes: association of retinal nerve fibre layer thickness with cognitive performance in a population-based study
Source: Brain Commun. 2021 Nov 8;3(4):fcab258. doi: 10.1093/braincomms/fcab258 (PMC8936429; doi:10.1093/braincomms/fcab258)
Supplement: fcab258_Supplementary_Data [file fcab258_Supplementary_Data.zip › Supplementary_Material_1-3.pdf]

Supporting Information

to

Reading cognition from the eyes – Association of retinal nerve fiber layer thickness with cognitive performance in a population-based study

Johanna Girbardt, MD[1,2], Tobias Luck, PhD[3], Jana Kynast, PhD[2], Francisca S. Rodriguez, PhD[4,5], Barbara Wicklein, Dipl.-Ing.[1], Kerstin Wirkner, PhD[6,1], Christoph Engel, MD[1,6], Christian Girbardt, MD[7], Mengyu Wang, PhD[8], Maryna Polyakova, PhD[2], A. Veronica Witte, PhD[2,6], Markus Loeffler, MD[1,6], Arno Villringer, MD[2,9,6], Steffi G. Riedel-Heller, MD[4,6], Matthias L. Schroeter, MD[2,9,6], Tobias Elze, PhD[8,6,], Franziska G. Rauscher, PhD[1,6;§,*].*

[1] Institute for Medical Informatics, Statistics and Epidemiology, Leipzig University, Leipzig, Germany
[2] Department of Neurology, Max Planck Institute for Human Cognitive and Brain Sciences, Leipzig
[3] Faculty of Applied Social Sciences, University of Applied Sciences Erfurt, Erfurt, Germany
[4] Germany Institute of Social Medicine, Occupational Health and Public Health (ISAP), Leipzig University, Leipzig, Germany
[5] German Centre for Neurodegenerative Diseases (DZNE), Research Group Psychosocial Epidemiology and Public Health, Greifswald, Germany
[6] Leipzig Research Centre for Civilization Diseases (LIFE), Leipzig University, Leipzig, Germany
[7] Department of Ophthalmology, Leipzig University Medical Center, Leipzig, Germany
[8] Schepens Eye Research Institute, Harvard Medical School, Boston, MA, USA
[9] Clinic of Cognitive Neurology, Leipzig University Medical Center, Leipzig, Germany

* These authors contributed equally

§ Corresponding author:
Dr. Franziska Rauscher (PhD)
Leipzig University
Institute for Medical Informatics, Statistics and Epidemiology
Härtelstraße 16-18
04107 Leipzig
Germany
E-Mail: franziska.rauscher@medizin.uni-leipzig.de

Supplementary Material 1 Exclusion criteria

Exclusion based on eye diseases which could possibly influence RNFLT

Two independent, experienced and clinically trained observers analyzed OCT scans and fundus images. In case of inter-observer differences, a consensus decision was reached to classify the participant's eye. Ophthalmic findings were graded based on current ophthalmological standards. Exclusion was based on these and on anamnestic information of previous eye disease. Diseases which typically affect both eyes like glaucoma or AMD lead to exclusion of the subject. For monocular conditions, e.g. amblyopia or most tumors, we excluded only the affected eye. For some categories we only excluded cases where RNFL is potentially affected, e.g. benign tumors of the anterior segment without impact on the optical axis were not excluded. 3,808 eyes were excluded due to relevant findings on fundus inspection which resulted in 2,591 excluded subjects.

Exclusion based on anamnestic information *

Main category	Eyes/ subjects	Sub-categories
**Glaucoma**	668/382	Glaucoma, elevated IOP
**Further optic nerve disorders**	39/24	Optic nerve disorders other than glaucoma
**AMD**	188/110	age-related macular degeneration (AMD)
**Further retinal disorders**	290/170	Retinal disorders other than AMD like diabetic retinopathy, optic disc drusen, retinal vascular occlusion
**Inflammatory disease**	16/11	Ocular toxoplasmosis, uveitis, other
**Substantial visual impairment**	382/234	anophthalmos, amblyopia, blindness or low vision, diplopia, color vision disorder, visual field defects, spatial vision deficits, other
**Trauma and orbital disease**	16/9	severe trauma, orbital disease
**Tumor**	6/4	relevant hemangioma, intraocular tumors, other relevant eye tumors

Exclusion based on conditions of the CNS which could possibly influence RNFLT *

Main category	Subjects	Sub-categories
**Tumor**	80	relevant brain tumors, chemotherapy in the past year, radiotherapy in close proximity to the CNS
**Stroke**	167	ischemic and hemorrhagic stroke based on medical history and MRI
**Parkinson's disease**	16	Parkinson's disease
**Multiple sclerosis**	10	multiple sclerosis
**Depression**	364	Sum score CES-D > 23
**Centrally acting drugs**	593	Opioids (ATC: N02A) , antiepileptics (N03), anti-Parkinson drugs (N04), antipsychotics (N05A), anxiolytics (N05B), hypnotics/sedatives (N05C), tricyclic antidepressants (N06AA), selective serotonin reuptake inhibitors (SSRI)(N06AB), phytotherapeutic antidepressants (N06AP), Serotonin and norepinephrine reuptake inhibitors (SNRI) (N06AX16, N06AX21)

* participants may fit more than one category

Supplementary Material 2
Neuropsychological Assessment

The LIFE-Adult study employs the following cognitive assessments. The performance and intention are shortly summarized.

CERAD: The CERAD test battery (The Consortium to Establish a Registry for Alzheimer's Disease) was developed to evaluate cognitive impairment associated with Alzheimer's disease (AD).[1,2] It has also been used to evaluate cognitive decline in general and other types of dementia.[3-5] It includes a clinical battery, a neuropsychological battery, and a neuropathological assessment. In this paper we focus on the selection of neuropsychological tests which were investigated in the LIFE study. We used the authorized German version of the CERAD-NP Plus (www.memoryclinic.ch).

Boston Naming Test (BNT, CERAD): This test is often used to assess confrontation naming, for example in individuals with aphasia or language problems caused by dementia.[6,7] In the 15-Item BNT, 15 pictured objects of different prevalence have to be named. The test also gives information about visual perception and word retrieval.

Mini-Mental State Examination (MMSE, CERAD): The MMSE is a widely used screening method to roughly estimate the overall cognitive status and accordingly assess the severity of dementia. Several areas of cognition are evaluated: orientation, immediate memory, attention, short delay recall (three words), working memory (forward and backward spelling), language, execution of oral commands and constructive abilities (copying). The maximum score is 30 points.[8,9]

Phonemic Fluency (CERAD – Plus): The examinees have to generate as many words as possible beginning with the same letter, here 'S', within one minute, excluding proper nouns and numbers. The task encompasses the verbal fluency more with a focus on strategy and less on semantic memory. Clinically, patients with Alzheimer's disease have been observed to be more impaired in semantic than in letter fluency tasks. [10]

Semantic Fluency Animals (CERAD): The ability to list animals within a time limit of one minute is tested. The task measures lexical knowledge and semantic memory organization, additionally to a certain extent executive functions. [11-13] It has been found that the result depends on the educational level.[14] PET studies give evidence that especially the temporal lobe is activated in semantic verbal fluency tests.[15]

Reading the Mind in the eye test (also called Eyes Task, RME): It is a test of theory of mind skills or in other words cognitive empathy. The emotional or mental state of a person is described only with photographs of their eyes, using a fixed-choice paradigm. The subject has to estimate what the presented person is feeling or thinking, finding the correct answer from four given emotion terms. There exists a gender difference with females outperforming males.[16] Here, we used the German version by Bölte.[17]

Trail Making Test (TMT, CERAD - Plus): The most widely used version of the TMT consists of two parts.[18] In Part A the subject is asked to draw a line between 25 consecutive numbers as fast as possible (e.g., 1 – 2 – 3 etc.). In Part B numbers and letters have to be linked alternately (e.g., 1 – A – 2 – B, etc.). Total time for completion of both tests in seconds is counted.[19] TMT-A predominantly provides information on the speed of cognitive processing and visual attention. TMT-B additionally tests higher cognitive skills like mental flexibility/ cognitive alternation and executive functions.[20-23] The TMT-B/A ratio removes the influence of motor speed and visual search which are measured by both TMT-A and TMT-B.

Multiple choice German vocabulary test "Mehrfachwahl-Wortschatz-Intelligenztest-B" (MWT-B): The instrument is widely used in German speaking countries. The subject has to choose one existing word out of five. The remaining four words are constructed. 37 items are presented in rising level of difficulty. The test is assumed to encompass the premorbid intelligence level as it is barely influenced by the actual efficiency.[24] However, some studies show an impairment to a certain content in patients with dementia and schizophrenia.[25,26] Especially semantic dementia comes with poor vocabulary performance.[27]

Stroop Test: The subject is asked to answer to a specific dimension of a question while suppressing an interfering dimension.[28] Cognitive inhibition is a main component of executive functions.[29] Here we used a computer-based color-word matching Stroop task like Zysset et al.[30] The task has two conditions. In the congruent condition, the ink color is the same as the written word. In the incongruent condition, it differed. For example, the subject has to name the ink color "red" of the written word "BLUE".

Visuoconstrucion Copy (CERAD): The subject is asked to copy four figures (circle, diamond, overlapping rectangles, three dimensional Necker cube). Here, the constructional praxis is tested.

Visuoconstrucion Recall (CERAD): The four figures have to be drawn from memory after a short interval. The participants are not informed when they first copy the items. Here, delayed figural (non-verbal) memory is tested.

Wordlist Learning (CERAD): Ten common nouns are visually presented to the participant and recalled immediately afterwards. There are three turns in which the words are presented in a different order, respectively. The task tests the verbal short-term memory and learning. Deficits can for example indicate an episodic memory dysfunction.

Wordlist Delayed Recall (CERAD): The ten nouns from the word list memory task have to be freely recalled after a 15 minutes interval. The mid-term verbal memory and free reproduction is tested.

Wordlist Recognition (CERAD): The participant has to recognize the ten learned nouns from a list of twenty words. The words are presented among 10 distractor words. With this task it is possible to differentiate between deficits in verbal delayed recall and recognition.

References

1. Welsh KA, Butters N, Mohs RC, et al. The Consortium to Establish a Registry for Alzheimer's Disease (CERAD). Part V. A normative study of the neuropsychological battery. *Neurology*. 1994;44(4):609-614.
2. Morris JC, Heyman A, Mohs RC, et al. The Consortium to Establish a Registry for Alzheimer's Disease (CERAD). Part I. Clinical and neuropsychological assessment of Alzheimer's disease. *Neurology*. 1989;39(9):1159-1165.
3. Cherrier MM, Mendez MF, Perryman KM, Pachana NA, Miller BL, Cummings JL. Frontotemporal dementia versus vascular dementia: differential features on mental status examination. *J Am Geriatr Soc*. 1997;45(5):579-583.
4. Karrasch M, Laatu S, Martikainen K, Marttila R. CERAD test performance and cognitive impairment in Parkinson's disease. *Acta Neurol Scand*. 2013;128(6):409-413. doi:10.1111/ane.12138
5. Schmid NS, Ehrensperger MM, Berres M, Beck IR, Monsch AU. The Extension of the German CERAD Neuropsychological Assessment Battery with Tests Assessing Subcortical, Executive and

Frontal Functions Improves Accuracy in Dementia Diagnosis. *DEE*. 2014;4(2):322-334. doi:10.1159/000357774

6. Kaplan E. *Boston Naming Test*. Lea & Febiger; 1983.
7. Rabin LA, Barr WB, Burton LA. Assessment practices of clinical neuropsychologists in the United States and Canada: A survey of INS, NAN, and APA Division 40 members. *Arch Clin Neuropsychol*. 2005;20(1):33-65. doi:10.1016/j.acn.2004.02.005
8. Folstein MF, Folstein SE, McHugh PR. "Mini-mental state". A practical method for grading the cognitive state of patients for the clinician. *J Psychiatr Res*. 1975;12(3):189-198.
9. Mungas D. In-office mental status testing: a practical guide. *Geriatrics*. 1991;46(7):54-8, 63, 66.
10. Laws KR, Duncan A, Gale TM. 'Normal' semantic-phonemic fluency discrepancy in Alzheimer's disease? A meta-analytic study. *Cortex*. 2010;46(5):595-601. doi:10.1016/j.cortex.2009.04.009
11. Bruyer R, Tuyumbu B. Fluence verbale et lésions du cortex cérébral: performances et types d'erreurs. *Encephale*. 1980;6(3):287-297.
12. Ardila A, Ostrosky-Solís F, Bernal B. Cognitive testing toward the future: The example of Semantic Verbal Fluency (ANIMALS). *International Journal of Psychology*. 2006;41(5):324-332. doi:10.1080/00207590500345542
13. Santos Nogueira D, Azevedo Reis E, Vieira A. Verbal Fluency Tasks: Effects of Age, Gender, and Education. *Folia Phoniatr Logop*. 2016;68(3):124-133. doi:10.1159/000450640
14. Ardila A, Ostrosky-Solis F, Rosselli M, Gomez C. Age-related cognitive decline during normal aging: the complex effect of education. *Arch Clin Neuropsychol*. 2000;15(6):495-513.
15. Warburton E, Wise RJ, Price CJ, et al. Noun and verb retrieval by normal subjects. Studies with PET. *Brain*. 1996;119 (Pt 1):159-179.
16. Baron-Cohen S, Wheelwright S, Hill J, Raste Y, Plumb I. The "Reading the Mind in the Eyes" Test revised version: a study with normal adults, and adults with Asperger syndrome or high-functioning autism. *J Child Psychol Psychiatry*. 2001;42(2):241-251.
17. Bölte S. *Reading Mind in the Eyes Test Für Erwachsene (Dt. Fassung) Von S. Baron-Cohen*. 2005.
18. Spreen O, Strauss E. *Compendium of Neuropsychological Tests, a.* 2nd ed. Oxford University Press; 1998.
19. Bowie CR, Harvey PD. Administration and interpretation of the Trail Making Test. *Nat Protoc*. 2006;1(5):2277-2281. doi:10.1038/nprot.2006.390
20. Lezak MD. *Neuropsychological Assessment.* 3. ed. Oxford University P; 1995.
21. Crowe SF. The differential contribution of mental tracking, cognitive flexibility, visual search, and motor speed to performance on parts A and B of the Trail Making Test. *J Clin Psychol*. 1998;54(5):585-591.
22. Arbuthnott K, Frank J. Trail making test, part B as a measure of executive control: validation using a set-switching paradigm. *J Clin Exp Neuropsychol*. 2000;22(4):518-528. doi:10.1076/1380-3395(200008)22:4;1-0;FT518
23. Varjacic A, Mantini D, Demeyere N, Gillebert CR. Neural signatures of Trail Making Test performance: Evidence from lesion-mapping and neuroimaging studies. *Neuropsychologia*. 2018;115:78-87. doi:10.1016/j.neuropsychologia.2018.03.031
24. Lehrl S. *Mehrfachwahl-Wortschatz-Intelligenztest: MWT-B.* 5., unveränd. Aufl. Spitta; 2005.
25. Wittorf A, Wiedemann G, Klingberg S. Mehrfachwahl-Wortschatz-Intelligenztest MWT bei Schizophrenie: Valides Maß der prämorbiden Intelligenz? *Psychiatr Prax*. 2014;41(2):95-100. doi:10.1055/s-0033-1349595
26. Kessler J, Fast K, Mielke R. Zur Problematik der prämorbiden Intelligenzdiagnostik mit dem MWT-B bei Patienten mit Alzheimer-Erkrankung. *Nervenarzt*. 1995;66(9):696-702.

27. Lezak MD, Howieson DB, Bigler ED, Tranel D. *Neuropsychological Assessment.* Fifth edition. Oxford University Press; 2012.
28. Stroop JR. Studies of interference in serial verbal reactions. *Journal of Experimental Psychology*. 1935;18(6):643-662. doi:10.1037/h0054651
29. Diamond A. Executive functions. *Annu Rev Psychol*. 2013;64:135-168. doi:10.1146/annurev-psych-113011-143750
30. Zysset S, Muller K, Lohmann G, von Cramon , D Y. Color-word matching stroop task: separating interference and response conflict. *Neuroimage*. 2001;13(1):29-36. doi:10.1006/nimg.2000.0665

Supplementary Material 3 Results

Mean values of RNFLT per sector

Segment	Sample A, mean RNFLT in µm	SD	Sample B, mean RNFLT in µm	SD
Global	97.3	9.3	97.4	9.3
Temporal	70.4	11.7	70.6	11.7
Temporal-superior	133.4	17.3	133.7	17.3
Temporal-inferior	141.6	6.18	107.3	22.6
Nasal	75	15.2	75.1	15.1
Nasal-superior	105.1	20.4	141.9	18.1
Nasal-inferior	107	22.5	105.3	20.5

Abbreviation: RNFLT, retinal nerve fiber layer

Age effect on cognition

Linear regression analysis to predict test specific cognitive performance from age (Sample B).

	β_0	β age	p	p adjusted
BNT	14.036	0.007	0.250	0.25
MMSE	31.334	-0.044	**<0.001**	**<0.001**
MWT-B	110.901	-0.102	**<0.001**	**<0.001**
Phonemic fluency	15.602	-0.039	0.086	0.091
RMET	27.317	-0.076	**<0.001**	**<0.001**
Semantic fluency	29.661	-0.098	**<0.001**	**<0.001**
Stroop neutral	803.187	15.665	**<0.001**	**<0.001**
Stroop incongruent	936.134	18.857	**<0.001**	**<0.001**
Stroop inc./neut.	1.158	0.001	0.034	**0.041**
TMT-A	5.143	0.536	**<0.001**	**<0.001**
TMT-B	1.669	1.468	**<0.001**	**<0.001**
TMT B/A	2.132	0.006	**<0.001**	**<0.001**
Visuoconstruction copy	11.351	-0.009	0.056	0.063
Visuoconstruction recall	14.339	-0.05	**<0.001**	**<0.001**
Word list learning	29.833	-0.123	**<0.001**	**<0.001**
Word list recall	10.933	-0.05	**<0.001**	**<0.001**
Word list recognition	20.227	-0.009	**<0.001**	**<0.001**

Legend: β_0 intercept, ß age: regression coefficient, p adjusted: Bonferroni corrected significance level false discovery rate (FDR); BNT, Boston naming test; MMSE, Mini-mental State Examination; MWT-B, Multiple choice German vocabulary test "Mehrfachwahl-Wortschatz-Intelligenztest-B"; RME, reading the mind in the eyes test; inc., incongruent; neut, neutral; TMT, Trail Making Test

RNFLT per sector sample A – Data corresponding to Figure 3 – regression coefficients

Test/RNFLT	G β	G p	T β	T p	TS β	TS p	TI β	TI p	N β	N p	NS β	NS p	NI β	NI p
BNT	-0.0068	0.97	0.22	0.36	0.039	0.91	0.17	0.65	-0.16	0.59	-0.5	0.25	0.09	0.84
MMSE	0.17	0.15	0.23	0.1	0.023	0.91	0.26	0.24	0.16	0.37	-0.18	0.49	0.4	0.12
MWT-B	-0.000049	0.99	0.045	**<0.001**	0.03	**0.035**	0.012	0.43	-0.035	**0.0044**	-0.056	**0.0011**	-0.01	0.57
Phonemic fluency 'S'	-0.076	0.11	0.097	0.095	-0.19	**0.034**	-0.031	0.73	-0.16	**0.025**	-0.08	0.44	-0.18	0.081
RME	-0.038	0.47	0.024	0.72	0.016	0.87	-0.026	0.79	-0.052	0.53	-0.16	0.18	-0.11	0.38
Semantic fluency	0.03	**0.026**	0.024	0.17	0.048	0.066	0.0021	0.94	0.018	0.42	0.038	0.21	0.057	0.082
Stroop neutral	-0.00072	**0.0012**	-0.00051	0.078	-0.00052	0.22	-0.00097	**0.028**	-0.00091	**0.014**	-0.00093	0.066	-0.00057	0.3
Stroop inc.	-0.00042	**0.0089**	-0.0002	0.33	-0.00043	0.17	-0.00051	0.11	-0.00058	**0.032**	-0.001	**0.0063**	-0.000045	0.91
Stroop inc./neut.	0.29	0.39	0.68	0.12	0.56	0.38	1.4	**0.035**	0.049	0.93	-2.1	**0.0062**	0.94	0.25
TMT-A	-0.034	**<0.001**	-0.032	**<0.001**	-0.057	**<0.001**	-0.048	**<0.001**	-0.024	**0.032**	-0.038	**0.014**	-0.019	0.25
TMT-B	-0.0065	**0.0026**	-0.006	**0.035**	-0.013	**0.0026**	-0.011	**0.0091**	-0.0019	0.59	-0.0069	0.16	-0.0058	0.28
TMT B/A	0.059	0.52	0.058	0.63	0.063	0.72	-0.038	0.84	0.18	0.24	0.11	0.61	-0.14	0.54
Visuoconstruction copy	0.24	0.28	0.23	0.43	-0.03	0.94	0.89	**0.042**	0.3	0.39	-0.34	0.5	0.39	0.44
Visuoconstruction recall	-0.023	0.77	0.15	0.12	0.077	0.6	0.19	0.19	-0.22	0.063	-0.2	0.24	-0.11	0.55
Wordlist learning	0.035	0.47	0.015	0.81	0.089	0.32	-0.0064	0.94	0.055	0.47	-0.036	0.74	0.073	0.52
Wordlist recall	-0.049	0.62	0.078	0.54	0.09	0.63	-0.16	0.42	-0.0098	0.95	-0.35	0.12	-0.12	0.61
Wordlist recognition	-0.16	0.44	0.24	0.37	-0.084	0.84	-0.56	0.18	-0.34	0.32	-0.27	0.58	-0.28	0.58

β: linear regression coefficient of the cognitive test score adjusted for age, sex, radius, education

p: p-values (uncorrected)

G: global, overall average; T: temporal; TS: temporal-superior; TI: temporal-inferior; N: nasal; NS: nasal-superior; NI: nasal-inferior

RNFLT per sector sample B - Data corresponding to Figure 3 – regression coefficients

Test/ RNFLT	G ß	G p	T β	T p	TS β	TS p	TI β	TI p	N β	N p	NS β	NS p	NI β	NI p
BNT	-0.0042	0.98	0.27	0.32	-0.0035	0.99	0.37	0.37	-0.33	0.32	-0.53	0.26	0.21	0.66
MMSE	0.13	0.33	0.19	0.24	-0.082	0.74	0.091	0.72	0.17	0.38	-0.25	0.39	0.5	0.082
MWT-B	0.0013	0.87	0.041	**<0.001**	0.022	0.18	0.0088	0.6	-0.023	0.1	-0.057	**0.0028**	-0.0046	0.82
Phonemic fluency 'S'	-0.072	0.17	0.13	0.066	-0.27	**0.0071**	-0.023	0.83	-0.16	0.058	-0.051	0.67	-0.18	0.14
RMET	-0.099	0.085	-0.0083	0.91	-0.091	0.4	-0.085	0.44	-0.14	0.12	-0.22	0.099	-0.13	0.34
Semantic fluency	0.035	**0.019**	0.024	0.22	0.049	0.089	0.023	0.44	0.026	0.3	0.012	0.73	0.084	**0.022**
Stroop neutral	-0.00078	**0.0019**	-0.00048	0.14	-0.00076	0.12	-0.00098	0.052	-0.001	**0.014**	-0.00079	0.17	-0.00078	0.21
Stroop inc.	-0.00046	**0.011**	-0.00024	0.32	-0.00072	0.04	-0.00051	0.16	-0.00061	**0.045**	-0.00091	**0.028**	5.70E-05	0.9
Stroop inc./neut.	0.31	0.37	0.64	0.16	0.43	0.52	1.5	**0.033**	0.12	0.84	-1.9	**0.014**	1	0.23
TMT-A	-0.037	**<0.001**	-0.028	**0.0068**	-0.055	**<0.001**	-0.043	**0.0064**	-0.033	**0.012**	-0.045	**0.012**	-0.032	0.095
TMT-B	-0.006	**0.016**	-0.0041	0.21	-0.009	0.063	-0.0073	0.14	-0.0034	0.41	-0.01	0.071	-0.0068	0.27
TMT B/A	0.11	0.28	0.1	0.46	0.19	0.33	0.085	0.68	0.18	0.29	0.048	0.84	3.10E-05	1
Wordlist learning	-0.00014	1	0.03	0.67	0.063	0.54	-0.019	0.86	-0.017	0.84	-0.12	0.34	0.045	0.73
Wordlist recall	-0.055	0.62	0.17	0.24	0.11	0.61	-0.073	0.74	-0.092	0.61	-0.48	0.062	-0.13	0.63
Wordlist recognition	-0.24	0.31	0.39	0.2	-0.14	0.75	-0.59	0.21	-0.42	0.26	-0.4	0.46	-0.75	0.18
Visuoconstruction copy	0.21	0.42	0.27	0.42	-0.023	0.96	0.89	0.085	0.35	0.39	-0.38	0.52	-0.0063	0.99
Visuoconstruction recall	-0.091	0.32	0.13	0.25	-0.06	0.73	0.19	0.3	-0.25	0.075	-0.29	0.15	-0.3	0.14

β: linear regression coefficient of the cognitive test score adjusted for age, sex, radius, education

p: p-values (uncorrected)

G: global, overall average; T: temporal; TS: temporal-superior; TI: temporal-inferior; N: nasal; NS: nasal-superior; NI: nasal-inferior

Data sample A corresponding to Figure 3 – regression coefficients

β: linear regression coefficient of the cognitive test score adjusted for age, sex, radius, education
p: p-values (**uncorrected**)

G: global, overall average; T: temporal; TS: temporal-superior; TI: temporal-inferior; N: nasal; NS: nasal-superior; NI: nasal-inferior

MMSE

sector	β_intercept	β_testscore	β_age	β_sex	β_radius	β_education	p_intercept	p_testscore	p_age	p_sex	p_radius	p_education
G	196.8111	0.1653	-0.2939	-1.8701	-48.9431	-0.0418	**<0.001**	0.1477	**<0.001**	**<0.001**	**<0.001**	0.7632
T	43.4542	0.2328	-0.1974	-2.6675	19.7094	-0.1860	**<0.001**	0.1026	**<0.001**	**<0.001**	**<0.001**	0.2836
TS	246.9824	0.0233	-0.4689	-0.6432	-50.2835	0.4050	**<0.001**	0.9142	**<0.001**	0.4067	**<0.001**	0.1231
TI	252.0169	0.2600	-0.5435	-4.5538	-47.7136	0.0011	**<0.001**	0.2379	**<0.001**	**<0.001**	**<0.001**	0.9966
N	205.8326	0.1588	-0.1648	-1.5665	-70.6939	-0.1770	**<0.001**	0.3702	**0.0123**	**0.0139**	**<0.001**	0.4117
NS	259.3651	-0.1768	-0.2644	0.9174	-76.0157	-0.2469	**<0.001**	0.4897	**0.0054**	0.3184	**<0.001**	0.4280
NI	318.5649	0.3973	-0.3515	-2.1422	-115.5328	0.2273	**<0.001**	0.1230	**<0.001**	**0.0206**	**<0.001**	0.4681

Wordlist learning

sector	β_intercept	β_testscore	β_age	β_sex	β_radius	β_education	p_intercept	p_testscore	p_age	p_sex	p_radius	p_education
G	190.0368	0.0346	-0.1624	-1.8619	-48.1890	0.0586	**<0.001**	0.4662	**<0.001**	**<0.001**	**<0.001**	0.5847
T	48.5623	0.0146	-0.0908	-2.6507	15.4774	0.0400	**<0.001**	0.8084	**<0.001**	**<0.001**	**<0.001**	0.7691
TS	219.9120	0.0889	-0.2563	-0.9186	-43.4846	0.3550	**<0.001**	0.3243	**<0.001**	0.1346	**<0.001**	0.0812
TI	248.1524	-0.0064	-0.3034	-4.7531	-50.8215	0.0904	**<0.001**	0.9445	**<0.001**	**<0.001**	**<0.001**	0.6644
N	202.7911	0.0548	-0.0943	-1.1579	-70.1777	-0.0542	**<0.001**	0.4710	**<0.001**	**0.0254**	**<0.001**	0.7521
NS	227.9297	-0.0361	-0.1874	0.5668	-63.9514	0.0672	**<0.001**	0.7378	**<0.001**	0.4396	**<0.001**	0.7821
NI	322.8844	0.0731	-0.1839	-2.1958	-118.2988	-0.0194	**<0.001**	0.5168	**<0.001**	**0.0043**	**<0.001**	0.9393

Wordlist recall

sector	β_intercept	β_testscore	β_age	β_sex	β_radius	β_education	p_intercept	p_testscore	p_age	p_sex	p_radius	p_education
G	191.4644	-0.0492	-0.1687	-1.9475	-48.1581	0.0821	**<0.001**	0.6212	**<0.001**	**<0.001**	**<0.001**	0.4396
T	48.2392	0.0779	-0.0888	-2.6214	15.4349	0.0342	**<0.001**	0.5378	**<0.001**	**<0.001**	**<0.001**	0.7998
TS	221.5441	0.0902	-0.2623	-0.9948	-43.5271	0.3809	**<0.001**	0.6331	**<0.001**	0.1037	**<0.001**	0.0590
TI	249.4504	-0.1574	-0.3101	-4.8476	-50.7336	0.1125	**<0.001**	0.4158	**<0.001**	**<0.001**	**<0.001**	0.5857
N	204.4122	-0.0098	-0.1011	-1.2483	-70.1671	-0.0278	**<0.001**	0.9510	**<0.001**	**0.0155**	**<0.001**	0.8700
NS	230.2188	-0.3511	-0.1998	0.3894	-63.7573	0.1068	**<0.001**	0.1198	**<0.001**	0.5937	**<0.001**	0.6575
NI	326.0657	-0.1216	-0.1981	-2.3883	-118.2237	0.0331	**<0.001**	0.6071	**<0.001**	**0.0018**	**<0.001**	0.8957

Wordlist recognition

sector	β_intercept	β_testscore	β_age	β_sex	β_radius	β_education	p_intercept	p_testscore	p_age	p_sex	p_radius	p_education
G	193.9845	-0.1633	-0.1669	-1.9843	-48.0297	0.0782	**<0.001**	0.4440	**<0.001**	**<0.001**	**<0.001**	0.4583
T	44.4956	0.2415	-0.0888	-2.6216	15.1626	0.0516	**<0.001**	0.3725	**<0.001**	**<0.001**	**<0.001**	0.6997
TS	223.9537	-0.0840	-0.2655	-1.0692	-43.4604	0.4005	**<0.001**	0.8356	**<0.001**	0.0762	**<0.001**	**0.0452**
TI	259.3592	-0.5560	-0.3068	-4.8307	-51.0482	0.1391	**<0.001**	0.1789	**<0.001**	**<0.001**	**<0.001**	0.4964
N	210.5525	-0.3384	-0.1041	-1.3760	-69.7508	-0.0383	**<0.001**	0.3204	**<0.001**	**0.0067**	**<0.001**	0.8200
NS	232.0234	-0.2667	-0.1852	0.4838	-63.7054	0.0259	**<0.001**	0.5811	**<0.001**	0.5019	**<0.001**	0.9135
NI	328.8110	-0.2825	-0.1927	-2.4669	-117.3124	0.0196	**<0.001**	0.5768	**<0.001**	**0.0011**	**<0.001**	0.9376

Visuoconstruction copy

sector	β_intercept	β_testscore	β_age	β_sex	β_radius	β_education	p_intercept	p_testscore	p_age	p_sex	p_radius	p_education
G	199.0877	0.2433	-0.2970	-1.9712	-48.9849	-0.0093	**<0.001**	0.2831	**<0.001**	**<0.001**	**<0.001**	0.9458
T	50.0305	0.2255	-0.2119	-2.6716	18.7470	-0.1337	**<0.001**	0.4254	**<0.001**	**<0.001**	**<0.001**	0.4352
TS	247.9194	-0.0296	-0.4741	-0.6936	-50.0929	0.4294	**<0.001**	0.9450	**<0.001**	0.3735	**<0.001**	0.0979
TI	250.5256	0.8901	-0.5481	-4.7509	-47.9764	0.0394	**<0.001**	**0.0420**	**<0.001**	**<0.001**	**<0.001**	0.8819
N	206.2760	0.3030	-0.1633	-1.6842	-70.2756	-0.1625	**<0.001**	0.3891	**0.0129**	**0.0085**	**<0.001**	0.4455
NS	256.2177	-0.3440	-0.2474	0.8827	-75.5287	-0.2710	**<0.001**	0.4978	**<0.001**	0.3391	**<0.001**	0.3777
NI	324.9806	0.3939	-0.3557	-2.4221	-115.0917	0.3021	**<0.001**	0.4413	**<0.001**	**0.0093**	**<0.001**	0.3295

Visuoconstruction recall

sector	β_intercept	β_testscore	β_age	β_sex	β_radius	β_education	p_intercept	p_testscore	p_age	p_sex	p_radius	p_education
G	202.2707	-0.0229	-0.3011	-1.9448	-49.0404	0.0005	**<0.001**	0.7676	**<0.001**	**<0.001**	**<0.001**	0.9970
T	50.3733	0.1496	-0.2011	-2.7874	18.7489	-0.1754	**<0.001**	0.1222	**<0.001**	**<0.001**	**<0.001**	0.3158
TS	247.3108	0.0772	-0.4712	-0.6986	-50.3580	0.3728	**<0.001**	0.5992	**<0.001**	0.3753	**<0.001**	0.1603
TI	258.8525	0.1947	-0.5390	-4.7228	-48.6273	-0.0340	**<0.001**	0.1948	**<0.001**	**<0.001**	**<0.001**	0.9003
N	211.9284	-0.2232	-0.1790	-1.5436	-70.0123	-0.0448	**<0.001**	0.0635	**0.0070**	**0.0167**	**<0.001**	0.8366
NS	254.7256	-0.2049	-0.2577	0.8840	-75.2455	-0.2234	**<0.001**	0.2370	**0.0071**	0.3412	**<0.001**	0.4752
NI	332.5400	-0.1056	-0.3802	-2.2779	-115.4715	0.3087	**<0.001**	0.5460	**<0.001**	**0.0152**	**<0.001**	0.3285

Phonemic fluency

sector	β_intercept	β_testscore	β_age	β_sex	β_radius	β_education	p_intercept	p_testscore	p_age	p_sex	p_radius	p_education
G	202.8040	-0.0756	-0.2940	-1.9952	-49.3920	0.0675	**<0.001**	0.1051	**<0.001**	**<0.001**	**<0.001**	0.6300
T	50.4664	0.0971	-0.2089	-2.5757	19.1903	-0.1933	**<0.001**	0.0952	**<0.001**	**<0.001**	**<0.001**	0.2694
TS	250.4551	-0.1862	-0.4733	-0.8976	-50.6966	0.5656	**<0.001**	**0.0344**	**<0.001**	0.2481	**<0.001**	**0.0326**
TI	262.6081	-0.0307	-0.5569	-4.6073	-49.1108	0.1132	**<0.001**	0.7338	**<0.001**	**<0.001**	**<0.001**	0.6765
N	211.8098	-0.1618	-0.1573	-1.7845	-71.0437	-0.0057	**<0.001**	**0.0253**	**0.0131**	**0.0052**	**<0.001**	0.9789
NS	252.8403	-0.0803	-0.2319	0.7887	-75.8927	-0.2075	**<0.001**	0.4421	**0.0113**	0.3927	**<0.001**	0.5091
NI	332.0866	-0.1837	-0.3590	-2.4585	-115.6945	0.4564	**<0.001**	0.0809	**<0.001**	**0.0082**	**<0.001**	0.1493

Reading the Mind in the Eyes

sector	β_intercept	β_testscore	β_age	β_sex	β_radius	β_education	p_intercept	p_testscore	p_age	p_sex	p_radius	p_education
G	190.9169	-0.0375	-0.1624	-1.7255	-48.3036	0.2710	**<0.001**	0.4696	**<0.001**	**<0.001**	**<0.001**	**0.0331**
T	47.6212	0.0239	-0.0817	-2.5072	15.0232	0.2543	**<0.001**	0.7163	**<0.001**	**<0.001**	**<0.001**	0.1145
TS	217.7319	0.0156	-0.2708	-0.8896	-41.4979	0.5875	**<0.001**	0.8729	**<0.001**	0.2116	**<0.001**	**0.0137**
TI	234.1786	-0.0261	-0.2893	-4.6685	-44.0128	0.4495	**<0.001**	0.7945	**<0.001**	**<0.001**	**<0.001**	0.0669
N	207.3268	-0.0517	-0.0925	-0.9132	-71.9466	0.1663	**<0.001**	0.5329	**<0.001**	0.1327	**<0.001**	0.4130
NS	234.1991	-0.1585	-0.2036	0.3184	-65.7760	0.2592	**<0.001**	0.1807	**<0.001**	0.7135	**<0.001**	0.3716
NI	333.1941	-0.1088	-0.1917	-1.7783	-121.8260	0.0372	**<0.001**	0.3822	**<0.001**	0.0512	**<0.001**	0.9029

Semantic fluency

sector	β_intercept	β_testscore	β_age	β_sex	β_radius	β_education	p_intercept	p_testscore	p_age	p_sex	p_radius	p_education
G	186.4310	0.0301	-0.1631	-1.2157	-45.9623	0.0552	**<0.001**	**0.0259**	**<0.001**	**<0.001**	**<0.001**	0.3520
T	54.5951	0.0242	-0.1098	-2.0131	11.6313	0.3968	**<0.001**	0.1702	**<0.001**	**<0.001**	**<0.001**	**<0.001**
TS	221.8686	0.0478	-0.2584	-0.1564	-43.5296	0.2124	**<0.001**	0.0659	**<0.001**	0.6298	**<0.001**	0.0633
TI	243.2961	0.0021	-0.3139	-3.2305	-48.3815	0.4106	**<0.001**	0.9368	**<0.001**	**<0.001**	**<0.001**	**<0.001**
N	193.5985	0.0181	-0.0859	-0.9182	-64.3840	-0.1993	**<0.001**	0.4191	**<0.001**	**0.0011**	**<0.001**	**0.0435**
NS	227.4124	0.0382	-0.1679	1.3880	-64.5854	-0.2968	**<0.001**	0.2134	**<0.001**	**0.0003**	**<0.001**	**0.0279**
NI	302.8976	0.0575	-0.1752	-1.8659	-105.7545	-0.2741	**<0.001**	0.0820	**<0.001**	**<0.001**	**<0.001**	0.0594

Boston Naming Test

sector	β_intercept	β_testscore	β_age	β_sex	β_radius	β_education	p_intercept	p_testscore	p_age	p_sex	p_radius	p_education
G	202.3314	-0.0068	-0.2953	-1.9354	-49.4396	0.0148	**<0.001**	0.9717	**<0.001**	**<0.001**	**<0.001**	0.9135
T	48.1938	0.2196	-0.2071	-2.7531	19.1884	-0.1278	**<0.001**	0.3616	**<0.001**	**<0.001**	**<0.001**	0.4515
TS	247.9219	0.0389	-0.4731	-0.7499	-50.6194	0.4310	**<0.001**	0.9149	**<0.001**	0.3406	**<0.001**	0.0934
TI	260.0964	0.1694	-0.5576	-4.6664	-49.1923	0.0901	**<0.001**	0.6497	**<0.001**	**<0.001**	**<0.001**	0.7320
N	212.9376	-0.1614	-0.1609	-1.5922	-71.1495	-0.1162	**<0.001**	0.5894	**0.0113**	**0.0138**	**<0.001**	0.5816
NS	259.6020	-0.5005	-0.2365	1.0631	-75.9747	-0.2545	**<0.001**	0.2467	**0.0099**	0.2547	**<0.001**	0.4035
NI	329.1776	0.0903	-0.3604	-2.3501	-115.7409	0.3249	**<0.001**	0.8357	**<0.001**	**0.0125**	**<0.001**	0.2899

TMT-A

sector	β_intercept	β_testscore	β_age	β_sex	β_radius	β_education	p_intercept	p_testscore	p_age	p_sex	p_radius	p_education
G	187.6226	-0.0341	-0.1470	-1.1778	-46.0756	0.0620	**<0.001**	**<0.001**	**<0.001**	**<0.001**	**<0.001**	0.2855
T	55.3125	-0.0316	-0.0939	-1.9855	11.6777	0.4017	**<0.001**	**<0.001**	**<0.001**	**<0.001**	**<0.001**	**<0.001**
TS	224.2272	-0.0575	-0.2315	-0.1215	-43.9163	0.2133	**<0.001**	**<0.001**	**<0.001**	0.7078	**<0.001**	0.0568
TI	244.1491	-0.0477	-0.2875	-3.1587	-48.6510	0.3836	**<0.001**	**<0.001**	**<0.001**	**<0.001**	**<0.001**	**<0.001**
N	194.3401	-0.0240	-0.0742	-0.8886	-64.4504	-0.2009	**<0.001**	**<0.001**	**<0.001**	**0.0015**	**<0.001**	**0.0377**
NS	229.2986	-0.0376	-0.1523	1.4290	-64.9130	-0.2841	**<0.001**	**<0.001**	**<0.001**	**<0.001**	**<0.001**	**0.0317**
NI	304.2833	-0.0191	-0.1695	-1.8157	-105.7061	-0.2223	**<0.001**	0.2472	**<0.001**	**<0.001**	**<0.001**	0.1191

TMT-B

sector	β_intercept	β_testscore	β_age	β_sex	β_radius	β_education	p_intercept	p_testscore	p_age	p_sex	p_radius	p_education
G	188.5167	-0.0065	-0.1576	-1.1696	-46.5845	0.0450	**<0.001**	**0.0026**	**<0.001**	**<0.001**	**<0.001**	0.4478
T	54.9637	-0.0060	-0.1019	-2.0056	11.8248	0.3862	**<0.001**	**0.0352**	**<0.001**	**<0.001**	**<0.001**	**<0.001**
TS	224.7561	-0.0126	-0.2441	-0.1128	-44.2660	0.1760	**<0.001**	**0.0026**	**<0.001**	0.7292	**<0.001**	0.1239
TI	245.0885	-0.0112	-0.2975	-3.1518	-49.2199	0.3544	**<0.001**	**0.0091**	**<0.001**	**<0.001**	**<0.001**	**0.0026**
N	195.8073	-0.0019	-0.0874	-0.8912	-65.2959	-0.1923	**<0.001**	0.5926	**<0.001**	**0.0015**	**<0.001**	0.0510
NS	230.5478	-0.0069	-0.1660	1.4713	-65.5408	-0.3215	**<0.001**	0.1604	**<0.001**	**<0.001**	**<0.001**	**0.0172**
NI	306.5028	-0.0058	-0.1756	-1.7626	-106.8441	-0.2417	**<0.001**	0.2782	**<0.001**	**<0.001**	**<0.001**	0.0964

Ratio TMT B/A

sector	β_intercept	β_testscore	β_age	β_sex	β_radius	β_education	p_intercept	p_testscore	p_age	p_sex	p_radius	p_education
G	187.6445	0.0587	-0.1673	-1.2044	-46.2889	0.0901	**<0.001**	0.5245	**<0.001**	**<0.001**	**<0.001**	0.1253
T	54.2030	0.0582	-0.1106	-2.0442	12.0688	0.4279	**<0.001**	0.6311	**<0.001**	**<0.001**	**<0.001**	**<0.001**
TS	223.3631	0.0634	-0.2625	-0.1870	-43.7756	0.2564	**<0.001**	0.7218	**<0.001**	0.5659	**<0.001**	**0.0239**
TI	243.9387	-0.0377	-0.3132	-3.2013	-48.7109	0.4190	**<0.001**	0.8372	**<0.001**	**<0.001**	**<0.001**	**<0.001**
N	194.9559	0.1789	-0.0910	-0.9075	-65.1187	-0.1620	**<0.001**	0.2431	**<0.001**	**0.0012**	**<0.001**	0.0973
NS	229.7023	0.1062	-0.1770	1.4285	-65.3133	-0.2722	**<0.001**	0.6131	**<0.001**	**<0.001**	**<0.001**	**0.0420**
NI	306.1405	-0.1388	-0.1830	-1.7659	-106.5495	-0.2186	**<0.001**	0.5396	**<0.001**	**<0.001**	**<0.001**	0.1294

MWT-B

sector	β_intercept	β_testscore	β_age	β_sex	β_radius	β_education	p_intercept	p_testscore	p_age	p_sex	p_radius	p_education
G	187.0979	0.0000	-0.1668	-1.2500	-45.8769	0.0833	**<0.001**	0.9947	**<0.001**	**<0.001**	**<0.001**	0.2028
T	51.5667	0.0453	-0.1094	-2.1252	11.4531	0.2261	**<0.001**	**<0.001**	**<0.001**	**<0.001**	**<0.001**	**0.0084**
TS	221.2928	0.0303	-0.2632	-0.2875	-43.9301	0.1209	**<0.001**	**0.0354**	**<0.001**	0.3793	**<0.001**	0.3381
TI	242.2715	0.0118	-0.3145	-3.2512	-48.3733	0.3764	**<0.001**	0.4259	**<0.001**	**<0.001**	**<0.001**	**0.0038**
N	196.8414	-0.0355	-0.0916	-0.8678	-64.1195	-0.0430	**<0.001**	**0.0044**	**<0.001**	**0.0021**	**<0.001**	0.6931
NS	232.4618	-0.0559	-0.1742	1.4348	-64.0716	-0.0388	**<0.001**	**0.0011**	**<0.001**	**<0.001**	**<0.001**	0.7949
NI	304.4560	-0.0103	-0.1813	-1.9002	-105.3682	-0.1472	**<0.001**	0.5740	**<0.001**	**<0.001**	**<0.001**	0.3594

Stroop neutral

sector	β_intercept	β_testscore	β_age	β_sex	β_radius	β_education	p_intercept	p_testscore	p_age	p_sex	p_radius	p_education
G	188.6881	-0.0007	-0.1537	-1.2709	-46.5219	0.0962	**<0.001**	**0.0012**	**<0.001**	**<0.001**	**<0.001**	0.1033
T	56.0525	-0.0005	-0.1056	-2.0126	11.4247	0.4263	**<0.001**	0.0781	**<0.001**	**<0.001**	**<0.001**	**<0.001**
TS	226.0158	-0.0005	-0.2569	-0.2197	-44.8854	0.2709	**<0.001**	0.2223	**<0.001**	0.5058	**<0.001**	**0.0174**
TI	244.0627	-0.0010	-0.2994	-3.3743	-48.3127	0.4261	**<0.001**	**0.0285**	**<0.001**	**<0.001**	**<0.001**	**<0.001**
N	195.9979	-0.0009	-0.0723	-1.0461	-65.1572	-0.1460	**<0.001**	**0.0135**	**<0.001**	**<0.001**	**<0.001**	0.1384
NS	230.5491	-0.0009	-0.1520	1.3553	-65.4799	-0.2802	**<0.001**	0.0658	**<0.001**	**<0.001**	**<0.001**	**0.0371**
NI	305.3056	-0.0006	-0.1652	-1.8067	-106.2527	-0.2150	**<0.001**	0.2987	**<0.001**	**<0.001**	**<0.001**	0.1381

Stroop incongruent

sector	β_intercept	β_testscore	β_age	β_sex	β_radius	β_education	p_intercept	p_testscore	p_age	p_sex	p_radius	p_education
G	188.5177	-0.0004	-0.1572	-1.2743	-46.5445	0.1036	**<0.001**	**0.0089**	**<0.001**	**<0.001**	**<0.001**	0.0791
T	55.7888	-0.0002	-0.1098	-2.0114	11.4336	0.4320	**<0.001**	0.3344	**<0.001**	**<0.001**	**<0.001**	**<0.001**
TS	225.9149	-0.0004	-0.2571	-0.2341	-44.8462	0.2756	**<0.001**	0.1673	**<0.001**	0.4786	**<0.001**	**0.0154**
TI	243.7382	-0.0005	-0.3049	-3.3864	-48.3247	0.4348	**<0.001**	0.1125	**<0.001**	**<0.001**	**<0.001**	**<0.001**
N	195.7992	-0.0006	-0.0761	-1.0486	-65.1710	-0.1360	**<0.001**	**0.0318**	**<0.001**	**<0.001**	**<0.001**	0.1669
NS	230.9929	-0.0010	-0.1480	1.3329	-65.6222	-0.2731	**<0.001**	**0.0063**	**<0.001**	**<0.001**	**<0.001**	**0.0418**
NI	304.8395	0.0000	-0.1751	-1.7841	-106.2951	-0.2072	**<0.001**	0.9103	**<0.001**	**<0.001**	**<0.001**	0.1526

Stroop incongruent/neutral

sector	β_intercept	β_testscore	β_age	β_sex	β_radius	β_education	p_intercept	p_testscore	p_age	p_sex	p_radius	p_education
G	187.7456	0.2870	-0.1654	-1.2410	-46.5317	0.1054	**<0.001**	0.3879	**<0.001**	**<0.001**	**<0.001**	0.0742
T	54.8138	0.6774	-0.1138	-1.9902	11.4301	0.4292	**<0.001**	0.1207	**<0.001**	**<0.001**	**<0.001**	**<0.001**
TS	224.8596	0.5641	-0.2655	-0.1936	-44.8564	0.2761	**<0.001**	0.3786	**<0.001**	0.5570	**<0.001**	**0.0152**
TI	241.6164	1.3964	-0.3152	-3.3296	-48.3162	0.4318	**<0.001**	**0.0346**	**<0.001**	**<0.001**	**<0.001**	**<0.001**
N	195.1452	0.0490	-0.0871	-1.0087	-65.1590	-0.1321	**<0.001**	0.9296	**<0.001**	**<0.001**	**<0.001**	0.1796
NS	232.3398	-2.0703	-0.1666	1.3782	-65.6068	-0.2535	**<0.001**	**0.0062**	**<0.001**	**<0.001**	**<0.001**	0.0589
NI	303.6993	0.9384	-0.1746	-1.7778	-106.2345	-0.2118	**<0.001**	0.2500	**<0.001**	**<0.001**	**<0.001**	0.1436

Data sample B corresponding to Figure 3 – regression coefficients

β: linear regression coefficient of the cognitive test score adjusted for age, sex, radius, education
p: p-values (**uncorrected**)

G: global, overall average; T: temporal; TS: temporal-superior; TI: temporal-inferior; N: nasal; NS: nasal-superior; NI: nasal-inferior

MMSE

sector	β_intercept	β_testscore	β_age	β_sex	β_radius	β_education	p_intercept	p_testscore	p_age	p_sex	p_radius	p_education
G	196.5863	0.1871	-0.3489	-1.9070	-46.7944	-0.0953	**<0.001**	0.1664	**<0.001**	**<0.001**	**<0.001**	0.5644
T	38.9569	0.2444	-0.1726	-2.3698	21.3184	-0.2641	**<0.001**	0.1487	**0.0082**	**<0.001**	**<0.001**	0.2018
TS	241.9661	0.0662	-0.4521	-0.9531	-48.2433	0.3207	**<0.001**	0.7939	**<0.001**	0.3152	**<0.001**	0.3009
TI	244.3207	0.2311	-0.5743	-4.3155	-41.1582	-0.1803	**<0.001**	0.3753	**<0.001**	**<0.001**	**<0.001**	0.5715
N	215.6417	0.1775	-0.2440	-1.8846	-73.5911	-0.1545	**<0.001**	0.3915	**0.0023**	**<0.001**	**<0.001**	0.5418
NS	268.4937	-0.2389	-0.4126	0.2020	-74.0872	-0.2298	**<0.001**	0.4317	**<0.001**	0.8590	**<0.001**	0.5361
NI	308.1996	0.5949	-0.5104	-1.7259	-106.3103	0.1511	**<0.001**	**0.0484**	**<0.001**	0.1259	**<0.001**	0.6816

Wordlist learning

sector	β_intercept	β_testscore	β_age	β_sex	β_radius	β_education	p_intercept	p_testscore	p_age	p_sex	p_radius	p_education
G	186.7714	0.0183	-0.1646	-1.8252	-45.6531	-0.0674	**<0.001**	0.7439	**<0.001**	**<0.001**	**<0.001**	0.5840
T	44.4553	0.0415	-0.0901	-2.3358	17.8446	-0.0926	**<0.001**	0.5602	**<0.001**	**<0.001**	**<0.001**	0.5537
TS	207.1847	0.1047	-0.2420	-0.8969	-35.8492	0.0413	**<0.001**	0.3206	**<0.001**	0.2038	**<0.001**	0.8584
TI	238.7159	0.0304	-0.2989	-4.4484	-45.1400	-0.2246	**<0.001**	0.7809	**<0.001**	**<0.001**	**<0.001**	0.3489
N	209.7248	-0.0039	-0.1059	-1.1593	-73.0726	-0.0807	**<0.001**	0.9652	**<0.001**	0.0526	**<0.001**	0.6807
NS	224.1892	-0.1382	-0.1951	0.0928	-60.2953	0.1669	**<0.001**	0.2796	**<0.001**	0.9136	**<0.001**	0.5520
NI	316.0447	0.0761	-0.1874	-2.4071	-113.6855	-0.1916	**<0.001**	0.5683	**<0.001**	**0.0071**	**<0.001**	0.5128

Wordlist recall

sector	β_intercept	β_testscore	β_age	β_sex	β_radius	β_education	p_intercept	p_testscore	p_age	p_sex	p_radius	p_education
G	187.7771	-0.0472	-0.1689	-1.8801	-45.6511	-0.0523	**<0.001**	0.6854	**<0.001**	**<0.001**	**<0.001**	0.6675
T	43.9940	0.1647	-0.0872	-2.2940	17.7957	-0.0985	**<0.001**	0.2665	**<0.001**	**<0.001**	**<0.001**	0.5242
TS	208.7006	0.1496	-0.2473	-0.9540	-35.9202	0.0656	**<0.001**	0.4950	**<0.001**	0.1746	**<0.001**	0.7746
TI	239.6772	-0.0083	-0.3028	-4.4966	-45.1505	-0.2099	**<0.001**	0.9710	**<0.001**	**<0.001**	**<0.001**	0.3762
N	210.4711	-0.0852	-0.1095	-1.2059	-73.0544	-0.0698	**<0.001**	0.6466	**<0.001**	**0.0429**	**<0.001**	0.7188
NS	225.9502	-0.5707	-0.2056	-0.0601	-60.1283	0.1898	**<0.001**	**0.0318**	**<0.001**	0.9437	**<0.001**	0.4937
NI	319.0066	-0.0757	-0.1999	-2.5615	-113.7010	-0.1468	**<0.001**	0.7851	**<0.001**	**0.0040**	**<0.001**	0.6124

Wordlist recognition

sector	β_intercept	β_testscore	β_age	β_sex	β_radius	β_education	p_intercept	p_testscore	p_age	p_sex	p_radius	p_education
G	191.9743	-0.2494	-0.1679	-1.9448	-45.4816	-0.0524	**<0.001**	0.3081	**<0.001**	**<0.001**	**<0.001**	0.6655
T	38.2880	0.3911	-0.0897	-2.3195	17.4350	-0.0738	**<0.001**	0.2079	**<0.001**	**<0.001**	**<0.001**	0.6314
TS	208.9361	0.0517	-0.2516	-1.0445	-35.8247	0.0883	**<0.001**	0.9105	**<0.001**	0.1326	**<0.001**	0.6982
TI	252.1060	-0.6037	-0.3074	-4.5670	-45.5161	-0.1420	**<0.001**	0.2044	**<0.001**	**<0.001**	**<0.001**	0.5465
N	217.3471	-0.4202	-0.1096	-1.3219	-72.5497	-0.0925	**<0.001**	0.2793	**<0.001**	**0.0244**	**<0.001**	0.6306
NS	232.3420	-0.6146	-0.1836	0.0656	-59.9887	0.0841	**<0.001**	0.2702	**<0.001**	0.9379	**<0.001**	0.7605
NI	332.4546	-0.8148	-0.2010	-2.7841	-112.5164	-0.1347	**<0.001**	0.1612	**<0.001**	**0.0016**	**<0.001**	0.6400

Visuoconstruction copy

sector	β_intercept	β_testscore	β_age	β_sex	β_radius	β_education	p_intercept	p_testscore	p_age	p_sex	p_radius	p_education
G	198.5972	0.2935	-0.3491	-1.9963	-46.7293	-0.0743	**<0.001**	0.2920	**<0.001**	**<0.001**	**<0.001**	0.6506
T	46.7833	0.1570	-0.1904	-2.3160	20.3725	-0.2203	**<0.001**	0.6522	**0.0035**	**<0.001**	**<0.001**	0.2833
TS	241.6031	0.1367	-0.4521	-0.9980	-47.7963	0.3264	**<0.001**	0.7935	**<0.001**	0.2982	**<0.001**	0.2890
TI	237.6375	1.0695	-0.5662	-4.5406	-40.4348	-0.1734	**<0.001**	**0.0460**	**<0.001**	**<0.001**	**<0.001**	0.5827
N	214.4375	0.4780	-0.2385	-2.0245	-73.1180	-0.1535	**<0.001**	0.2626	**0.0028**	**0.0099**	**<0.001**	0.5415
NS	259.1180	-0.0892	-0.3777	0.0760	-73.1532	-0.2938	**<0.001**	0.8866	**0.0013**	0.9473	**<0.001**	0.4254
NI	327.3036	-0.0422	-0.5298	-1.8738	-106.8406	0.2753	**<0.001**	0.9459	**<0.001**	0.1006	**<0.001**	0.4519

Visuoconstruction recall

sector	β_intercept	β_testscore	β_age	β_sex	β_radius	β_education	p_intercept	p_testscore	p_age	p_sex	p_radius	p_education
G	202.5448	-0.0534	-0.3520	-1.8455	-46.8464	-0.0542	**<0.001**	0.5829	**<0.001**	**<0.001**	**<0.001**	0.7466
T	45.6575	0.1292	-0.1765	-2.4980	20.7552	-0.2411	**<0.001**	0.2868	**0.0073**	**<0.001**	**<0.001**	0.2493
TS	243.8781	0.0021	-0.4593	-0.9450	-47.9992	0.3151	**<0.001**	0.9908	**<0.001**	0.3295	**<0.001**	0.3171
TI	247.6259	0.2434	-0.5598	-4.4163	-41.1851	-0.2523	**<0.001**	0.1945	**<0.001**	**<0.001**	**<0.001**	0.4355
N	221.5407	-0.2026	-0.2460	-1.7017	-73.0601	-0.0548	**<0.001**	0.1733	**0.0023**	**0.0312**	**<0.001**	0.8310
NS	262.8464	-0.3020	-0.3960	0.4449	-73.6850	-0.1854	**<0.001**	0.1661	**<0.001**	0.7005	**<0.001**	0.6221
NI	330.8979	-0.2058	-0.5468	-1.4983	-107.2747	0.2530	**<0.001**	0.3420	**<0.001**	0.1924	**<0.001**	0.4983

Phonemic fluency 'S'

sector	β_intercept	β_testscore	β_age	β_sex	β_radius	β_education	p_intercept	p_testscore	p_age	p_sex	p_radius	p_education
G	202.3977	-0.0634	-0.3386	-1.9640	-47.2692	-0.0022	**<0.001**	0.2748	**<0.001**	**<0.001**	**<0.001**	0.9894
T	45.6274	0.1384	-0.1847	-2.1811	21.0091	-0.3256	**<0.001**	0.0567	**0.0029**	**<0.001**	**<0.001**	0.1235
TS	246.7374	-0.2270	-0.4541	-1.2251	-48.5670	0.5105	**<0.001**	**0.0369**	**<0.001**	0.1986	**<0.001**	0.1068
TI	251.1971	0.0737	-0.5708	-4.1415	-42.0903	-0.1911	**<0.001**	0.5105	**<0.001**	**<0.001**	**<0.001**	0.5579
N	221.4639	-0.1495	-0.2239	-2.0478	-74.1350	0.0081	**<0.001**	0.0929	**0.0032**	**0.0087**	**<0.001**	0.9751
NS	258.4498	-0.1387	-0.3482	0.0240	-73.9189	-0.1401	**<0.001**	0.2882	**0.0018**	0.9833	**<0.001**	0.7125
NI	328.4876	-0.2110	-0.5123	-1.9774	-107.3444	0.4367	**<0.001**	0.1033	**<0.001**	0.0816	**<0.001**	0.2467

Reading the Mind in the Eyes Test

sector	β_intercept	β_testscore	β_age	β_sex	β_radius	β_education	p_intercept	p_testscore	p_age	p_sex	p_radius	p_education
G	191.9125	-0.1015	-0.1706	-1.5440	-47.6372	0.2143	**<0.001**	0.0872	**<0.001**	**<0.001**	**<0.001**	0.1401
T	49.2821	-0.0186	-0.0945	-2.1518	15.1194	0.2385	**<0.001**	0.8009	**<0.001**	**<0.001**	**0.0015**	0.1871
TS	209.7831	-0.0812	-0.2815	-0.9231	-34.6331	0.3550	**<0.001**	0.4624	**<0.001**	0.2598	**<0.001**	0.1895
TI	238.0583	-0.0959	-0.3075	-4.0843	-44.3437	0.2829	**<0.001**	0.3996	**<0.001**	**<0.001**	**<0.001**	0.3103
N	215.4920	-0.1393	-0.0978	-0.6522	-75.4301	0.1328	**<0.001**	0.1393	**<0.001**	0.3504	**<0.001**	0.5647
NS	227.0001	-0.2389	-0.2041	0.2055	-60.8711	0.3758	**<0.001**	0.0780	**<0.001**	0.8379	**<0.001**	0.2576
NI	332.7637	-0.1215	-0.1931	-2.0473	-120.9704	-0.0352	**<0.001**	0.3935	**<0.001**	0.0525	**<0.001**	0.9195

Semantic fluency

sector	β_intercept	β_testscore	β_age	β_sex	β_radius	β_education	p_intercept	p_testscore	p_age	p_sex	p_radius	p_education
G	184.8615	0.0324	-0.1595	-1.0735	-45.1703	0.0522	**<0.001**	**0.0317**	**<0.001**	**<0.001**	**<0.001**	0.4282
T	51.5394	0.0170	-0.1042	-1.9562	13.3470	0.3906	**<0.001**	0.3881	**<0.001**	**<0.001**	**<0.001**	**<0.001**
TS	218.8107	0.0424	-0.2417	-0.2555	-41.9287	0.1594	**<0.001**	0.1441	**<0.001**	0.4797	**<0.001**	0.2091
TI	239.3660	0.0249	-0.2997	-3.0933	-46.6478	0.3433	**<0.001**	0.4069	**<0.001**	**<0.001**	**<0.001**	**0.0089**
N	195.1564	0.0312	-0.0893	-0.7466	-65.3483	-0.2065	**<0.001**	0.2125	**<0.001**	**0.0167**	**<0.001**	0.0592
NS	224.3468	0.0012	-0.1716	1.5282	-62.5847	-0.1467	**<0.001**	0.9725	**<0.001**	**<0.001**	**<0.001**	0.3295
NI	303.5849	0.0821	-0.1776	-1.3510	-106.3353	-0.3015	**<0.001**	**0.0267**	**<0.001**	**0.0034**	**<0.001**	0.0629

Boston Naming Test

sector	β_intercept	β_testscore	β_age	β_sex	β_radius	β_education	p_intercept	p_testscore	p_age	p_sex	p_radius	p_education
G	203.4918	-0.0840	-0.3406	-1.8915	-47.4863	-0.0468	**<0.001**	0.7046	**<0.001**	**<0.001**	**<0.001**	0.7742
T	45.2318	0.1240	-0.1862	-2.3712	21.0661	-0.2215	**<0.001**	0.6547	**0.0027**	**<0.001**	**<0.001**	0.2776
TS	248.0466	-0.2313	-0.4530	-0.9150	-48.7556	0.3415	**<0.001**	0.5773	**<0.001**	0.3414	**<0.001**	0.2636
TI	251.5824	0.0911	-0.5767	-4.2999	-42.3963	-0.1303	**<0.001**	0.8312	**<0.001**	**<0.001**	**<0.001**	0.6786
N	224.1620	-0.2264	-0.2269	-1.8505	-74.5506	-0.0983	**<0.001**	0.5044	**0.0028**	**0.0186**	**<0.001**	0.6935
NS	264.9025	-0.5448	-0.3458	0.3850	-74.0528	-0.2414	**<0.001**	0.2741	**0.0019**	0.7386	**<0.001**	0.5101
NI	324.7862	0.1718	-0.5155	-1.8782	-107.6810	0.2805	**<0.001**	0.7281	**<0.001**	0.1009	**<0.001**	0.4403

TMT-A

sector	β_intercept	β_testscore	β_age	β_sex	β_radius	β_education	p_intercept	p_testscore	p_age	p_sex	p_radius	p_education
G	186.0666	-0.0363	-0.1430	-1.0336	-45.2579	0.0667	**<0.001**	**<0.001**	**<0.001**	**<0.001**	**<0.001**	0.3005
T	52.1698	-0.0262	-0.0909	-1.9215	13.3056	0.3963	**<0.001**	**0.0123**	**<0.001**	**<0.001**	**<0.001**	**<0.001**
TS	220.7794	-0.0562	-0.2161	-0.2203	-42.1813	0.1648	**<0.001**	**0.0003**	**<0.001**	0.5418	**<0.001**	0.1841
TI	240.1856	-0.0381	-0.2809	-3.0453	-46.6316	0.3512	**<0.001**	**0.0170**	**<0.001**	**<0.001**	**<0.001**	**0.0062**
N	196.3038	-0.0327	-0.0746	-0.7153	-65.4349	-0.1935	**<0.001**	**0.0141**	**<0.001**	**0.0216**	**<0.001**	0.0706
NS	225.7172	-0.0460	-0.1493	1.5872	-63.0987	-0.1680	**<0.001**	**0.0122**	**<0.001**	**<0.001**	**<0.001**	0.2537
NI	305.3245	-0.0332	-0.1669	-1.2960	-106.0905	-0.2271	**<0.001**	0.0929	**<0.001**	**0.0050**	**<0.001**	0.1522

TMT-B

sector	β_intercept	β_testscore	β_age	β_sex	β_radius	β_education	p_intercept	p_testscore	p_age	p_sex	p_radius	p_education
G	186.3880	-0.0067	-0.1536	-1.0146	-45.4684	0.0504	**<0.001**	**0.0089**	**<0.001**	**<0.001**	**<0.001**	0.4422
T	52.1612	-0.0038	-0.0996	-1.9415	13.2662	0.3908	**<0.001**	0.2564	**<0.001**	**<0.001**	**<0.001**	**<0.001**
TS	221.3618	-0.0116	-0.2296	-0.2038	-42.5588	0.1325	**<0.001**	**0.0190**	**<0.001**	0.5742	**<0.001**	0.2944
TI	241.1798	-0.0091	-0.2877	-3.0618	-47.2201	0.3218	**<0.001**	0.0734	**<0.001**	**<0.001**	**<0.001**	**0.0139**
N	196.0591	-0.0039	-0.0873	-0.6942	-65.3775	-0.1911	**<0.001**	0.3542	**<0.001**	**0.0264**	**<0.001**	0.0794
NS	225.9320	-0.0102	-0.1614	1.6678	-63.1538	-0.2222	**<0.001**	0.0817	**<0.001**	**<0.001**	**<0.001**	0.1380
NI	306.6350	-0.0074	-0.1774	-1.2284	-106.8025	-0.2355	**<0.001**	0.2412	**<0.001**	**0.0080**	**<0.001**	0.1441

Ratio TMT B/A

sector	β_intercept	β_testscore	β_age	β_sex	β_radius	β_education	p_intercept	p_testscore	p_age	p_sex	p_radius	p_education
G	185.3711	0.0833	-0.1632	-1.0522	-45.1259	0.0939	**<0.001**	0.4279	**<0.001**	**<0.001**	**<0.001**	0.1499
T	51.3936	0.0982	-0.1051	-1.9689	13.4913	0.4208	**<0.001**	0.4743	**<0.001**	**<0.001**	**<0.001**	**<0.001**
TS	219.7931	0.1148	-0.2461	-0.2735	-42.0182	0.2027	**<0.001**	0.5706	**<0.001**	0.4504	**<0.001**	0.1065
TI	240.0308	-0.0185	-0.2998	-3.0967	-46.7063	0.3708	**<0.001**	0.9296	**<0.001**	**<0.001**	**<0.001**	**0.0043**
N	195.0781	0.1558	-0.0934	-0.7202	-65.1215	-0.1552	**<0.001**	0.3717	**<0.001**	**0.0211**	**<0.001**	0.1516
NS	224.9926	0.0680	-0.1761	1.6012	-62.8590	-0.1659	**<0.001**	0.7769	**<0.001**	**<0.001**	**<0.001**	0.2653
NI	305.6296	-0.0025	-0.1872	-1.2521	-106.3691	-0.1942	**<0.001**	0.9923	**<0.001**	**0.0068**	**<0.001**	0.2255

MWT-B

sector	β_intercept	β_testscore	β_age	β_sex	β_radius	β_education	p_intercept	p_testscore	p_age	p_sex	p_radius	p_education
G	185.2466	0.0037	-0.1623	-1.1188	-45.1245	0.0766	**<0.001**	0.6671	**<0.001**	**<0.001**	**<0.001**	0.2924
T	48.7635	0.0401	-0.1030	-2.0281	13.1300	0.2438	**<0.001**	**<0.001**	**<0.001**	**<0.001**	**<0.001**	**0.0101**
TS	218.2534	0.0272	-0.2434	-0.4023	-42.3522	0.0837	**<0.001**	0.0978	**<0.001**	0.2689	**<0.001**	0.5499
TI	238.0532	0.0219	-0.3004	-3.1401	-46.7115	0.2968	**<0.001**	0.1972	**<0.001**	**<0.001**	**<0.001**	**0.0402**
N	197.8501	-0.0203	-0.0954	-0.7643	-65.3530	-0.0892	**<0.001**	0.1519	**<0.001**	**0.0149**	**<0.001**	0.4598
NS	228.3330	-0.0604	-0.1739	1.6211	-61.7978	0.0951	**<0.001**	**0.0020**	**<0.001**	**<0.001**	**<0.001**	0.5666
NI	304.7384	-0.0031	-0.1843	-1.4242	-105.7967	-0.1642	**<0.001**	0.8822	**<0.001**	**0.0022**	**<0.001**	0.3588

Stroop neutral

sector	β_intercept	β_testscore	β_age	β_sex	β_radius	β_education	p_intercept	p_testscore	p_age	p_sex	p_radius	p_education
G	187.0093	-0.0008	-0.1491	-1.1119	-45.5827	0.0979	**<0.001**	**0.0013**	**<0.001**	**<0.001**	**<0.001**	0.1358
T	53.4999	-0.0006	-0.0985	-1.9319	12.8201	0.4218	**<0.001**	0.0533	**<0.001**	**<0.001**	**<0.001**	**<0.001**
TS	222.3991	-0.0010	-0.2342	-0.2908	-42.8119	0.2100	**<0.001**	0.0538	**<0.001**	0.4284	**<0.001**	0.0964
TI	241.0633	-0.0014	-0.2815	-3.2714	-46.5186	0.3700	**<0.001**	**0.0067**	**<0.001**	**<0.001**	**<0.001**	**0.0047**
N	197.6085	-0.0009	-0.0765	-0.8455	-65.9761	-0.1364	**<0.001**	**0.0278**	**<0.001**	**0.0077**	**<0.001**	0.2110
NS	225.5214	-0.0005	-0.1603	1.5432	-63.1855	-0.1437	**<0.001**	0.4412	**<0.001**	**<0.001**	**<0.001**	0.3366
NI	305.4320	-0.0008	-0.1654	-1.3047	-106.0728	-0.2324	**<0.001**	0.2304	**<0.001**	**0.0054**	**<0.001**	0.1496

Stroop inc.

sector	β_intercept	β_testscore	β_age	β_sex	β_radius	β_education	p_intercept	p_testscore	p_age	p_sex	p_radius	p_education
G	186.7513	-0.0005	-0.1529	-1.1142	-45.5810	0.1067	**<0.001**	**0.0089**	**<0.001**	**<0.001**	**<0.001**	0.1036
T	53.2503	-0.0004	-0.1014	-1.9369	12.8482	0.4285	**<0.001**	0.1154	**<0.001**	**<0.001**	**<0.001**	**<0.001**
TS	222.2614	-0.0008	-0.2334	-0.3171	-42.7508	0.2179	**<0.001**	**0.0203**	**<0.001**	0.3882	**<0.001**	0.0840
TI	240.4066	-0.0007	-0.2898	-3.2760	-46.4521	0.3851	**<0.001**	0.0564	**<0.001**	**<0.001**	**<0.001**	**0.0032**
N	197.3268	-0.0005	-0.0813	-0.8449	-65.9910	-0.1262	**<0.001**	0.0848	**<0.001**	**0.0078**	**<0.001**	0.2466
NS	225.9655	-0.0007	-0.1544	1.5251	-63.2695	-0.1413	**<0.001**	0.0991	**<0.001**	**<0.001**	**<0.001**	0.3437
NI	304.7664	0.0001	-0.1789	-1.2677	-106.1244	-0.2203	**<0.001**	0.8522	**<0.001**	**0.0069**	**<0.001**	0.1714

Stroop inc./neut.

sector	β_intercept	β_testscore	β_age	β_sex	β_radius	β_education	p_intercept	p_testscore	p_age	p_sex	p_radius	p_education
G	185.9582	0.3160	-0.1623	-1.0766	-45.6078	0.1086	**<0.001**	0.3631	**<0.001**	**<0.001**	**<0.001**	0.0977
T	52.2816	0.5686	-0.1090	-1.9018	12.8177	0.4284	**<0.001**	0.2092	**<0.001**	**<0.001**	**<0.001**	**<0.001**
TS	221.0728	0.4578	-0.2494	-0.2494	-42.8358	0.2218	**<0.001**	0.4932	**<0.001**	0.4966	**<0.001**	0.0787
TI	238.0031	1.5827	-0.3042	-3.2042	-46.5042	0.3821	**<0.001**	**0.0222**	**<0.001**	**<0.001**	**<0.001**	**0.0035**
N	196.7015	0.1272	-0.0914	-0.8071	-66.0170	-0.1230	**<0.001**	0.8255	**<0.001**	**0.0108**	**<0.001**	0.2590
NS	227.5293	-1.9204	-0.1665	1.5467	-63.3115	-0.1262	**<0.001**	**0.0152**	**<0.001**	**<0.001**	**<0.001**	0.3979
NI	303.5526	1.0335	-0.1778	-1.2668	-106.0558	-0.2268	**<0.001**	0.2257	**<0.001**	**0.0068**	**<0.001**	0.1592
